# Mediator kinase inhibitors suppress triple-negative breast cancer growth and extend tumor suppression by mTOR and AKT inhibitors

**DOI:** 10.1073/pnas.2414501121

**Published:** 2024-11-14

**Authors:** Xiaokai Ding, Jiaxin Liang, Amanda C. Sharko, Thomas A. Hilimire, Jing Li, Jürgen Loskutov, Zachary T. Mack, Hao Ji, Gary P. Schools, Chao Cai, Elena N. Pugacheva, Mengqian Chen, Igor B. Roninson, Eugenia V. Broude

**Affiliations:** ^a^Department of Drug Discovery and Biomedical Sciences, College of Pharmacy, University of South Carolina, Columbia, SC 29208; ^b^Senex Biotechnology, Inc., Columbia, SC 29208; ^c^Department of Biochemistry and Molecular Medicine, West Virginia University Cancer Institute, School of Medicine, Morgantown, WV 26506; ^d^Department of Clinical Pharmacy and Outcomes Sciences, College of Pharmacy, University of South Carolina, Columbia, SC 29208

**Keywords:** triple negative breast cancer, CDK8, mediator kinase, mTOR, metastasis

## Abstract

Triple-negative breast cancers (TNBC) are treated primarily by chemotherapy and lack clinically validated therapeutic targets. In particular, inhibitors of the PI3K/AKT/mTOR pathway, abnormally activated in many breast cancers, failed to achieve clinical efficacy in TNBC due to the development of adaptive drug resistance, which is largely driven by the transcriptomic plasticity of TNBC. Expression of CDK8/19 Mediator kinases that control transcriptional reprogramming correlates with relapse-free survival and treatment failure in breast cancer patients, including TNBC. We now investigated how CDK8/19 inhibitors affect the growth of TNBC tumors and their response to mTOR and AKT inhibitors. In contrast to the effects of most anticancer drugs, all the tested human TNBC models (including patient-derived xenografts) responded to CDK8/19 inhibitors in vivo even when they did not respond in vitro. Furthermore, CDK8/19 inhibition extended the host survival of established lung metastases in a murine TNBC model, where the primary tumors were not significantly affected. CDK8/19 inhibitors synergized with an mTORC1 inhibitor everolimus and a pan-AKT inhibitor capivasertib in vitro and strongly potentiated these drugs in long-term in vivo studies. Transcriptomic analysis of tumors that responded or became adapted to everolimus revealed that drug adaptation in vivo was associated with major transcriptional changes in both tumor and stromal cells. Combining everolimus with a CDK8/19 inhibitor counteracted many of these changes and induced combination-specific effects on the expression of multiple genes that affect tumor growth. These results warrant the exploration of CDK8/19 Mediator kinase inhibitors as a new type of drugs for TNBC therapy.

## Significance

Transcriptomic plasticity of TNBC promotes metastatic growth and renders these cancers resistant to targeted therapies. Inhibition of CDK8/19 Mediator kinases, pleiotropic regulators of transcriptional reprogramming, inhibits the growth of TNBC tumors, extends the survival of metastatic disease, and strongly enhances tumor suppression by targeted drugs in vivo, suggesting that CDK8/19 inhibitors may achieve a transformative impact on TNBC therapy.

Breast cancers (BrCa) that are negative for estrogen receptor (ER), progesterone receptor, and HER2 are broadly classified as triple-negative breast cancer (TNBC). TNBCs are highly metastatic and lack clinically validated therapeutic targets; their treatment is still based largely on conventional chemotherapy, which is toxic and rarely curative. Many targeted drugs and immunotherapies are undergoing clinical trials but have not yet shown a clear benefit for TNBC, except for PARP and checkpoint inhibitors that benefit a small number of patients (1, 2). In particular, while the PI3K/AKT/mTOR signaling pathway is overactivated in at least half of TNBCs, inhibitors of this pathway have not shown clinical efficacy in TNBC trials (3). The key obstacle to achieving sustained therapeutic efficacy is the development of treatment resistance. It is now understood that the transcriptomic plasticity of tumor cells promotes therapy resistance via nongenetic (transcriptional) adaptation to treatment (4). The transcriptomic plasticity also enables the metastatic growth and its associated resistance of metastatic cells to different therapies (5). As a result, many drugs, while efficacious against primary tumors, have no effect in a metastatic setting (6). A therapeutic strategy aimed at transcriptomic plasticity per se could have a critical impact on the treatment of metastatic disease and the prevention of the adaptive drug resistance.

A novel class of anticancer drugs under clinical development target CDK8 and CDK19 Mediator kinases (clinicaltrials.gov NCT03065010, NCT04021368, NCT05052255, NCT05300438). CDK8 and its paralog CDK19 are alternative enzymatic components of the kinase module that binds to and regulates the transcriptional Mediator complex. This module also includes Cyclin C (CCNC, the binding partner of CDK8/19) as well as proteins MED12 and MED13 (7). Mediator kinases regulate transcription both positively, by potentiating signal-induced gene expression, and negatively, via posttranscriptional downregulation of Mediator complex subunits (8). CDK8/19 have been identified as broad-spectrum regulators of transcriptional reprogramming (8–11). By suppressing transcriptional reprogramming, CDK8/19 inhibitors (CDK8/19i) showed a unique ability to suppress the growth of metastases preferentially to primary tumors, as reported for colon cancer (12). In addition to their effects on tumor cells, CDK8/19i suppress tumor-promoting paracrine activities of stromal fibroblasts (13) and stimulate tumor surveillance by NK cells (14) and effector T cells (15). CDK8/19i were also found to prevent or reverse resistance to different classes of anticancer agents (13, 16–18).

In the context of BrCa, CDK8/19i were found to inhibit tumor growth, prevent the development of estrogen independence and potentiate antiestrogens in ER-positive BrCa (19). CDK8/19i also suppress tumor growth and prevent and reverse resistance to HER2-targeting drugs in HER2-positive BrCa (20). In the case of TNBC, it was reported that a CDK8/19i inhibited in vitro proliferation of a single TNBC cell line (21), but another study concluded that only CDK8 knockdown but not CDK8/19i exerted anticancer effects suggesting that such effects were kinase-independent (22). However, we have found in the present study that different selective Mediator kinase inhibitors suppress the growth of TNBC tumors, extend the survival of metastatic disease, and potentiate drugs targeting mTOR and AKT, preventing, at least in some cases, the tumor adaptation to therapy. These results warrant the exploration of CDK8/19i as a new type of drugs for TNBC therapy.

## Results

### CDK8/CDK19/CCNC Correlations with Survival and Therapy Failure in TNBC Patients.

**Fig. 1.**
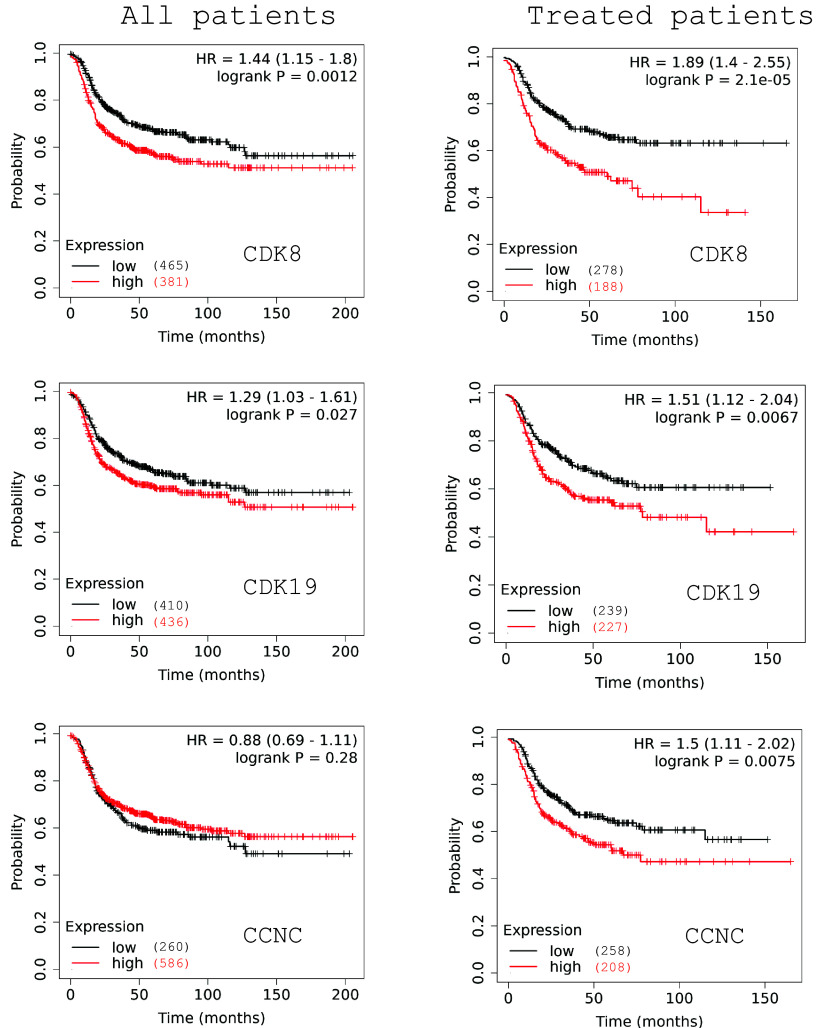
Correlations of CDK8, CDK19, and CCNC RNA expression with Relapse Free Survival in microarray data (KMplot.com) from 846 patients classified as ER- and HER2- according to microarray signals (*Left*) and from 466 patients from the same group who received therapy after sample collection (*Right*).

We have used KMplotter (23) to analyze survival correlations of gene expression of CDK8, CDK19, and their binding partner CCNC using microarray data from 846 BrCa patients that were negative for ER and HER2 (an approximation of TNBC). The expression of CDK8 and CDK19 (but not of CCNC) correlated in such patients with shorter relapse-free survival (RFS), but the RFS correlations for CDK8 and CDK19 became much stronger and also emerged for CCNC when the analysis was restricted to 466 patients known to have received systemic therapy following sample collection (Fig. 1). Hence, Mediator kinase expression impacts the survival of TNBC patients most likely by influencing the outcome of therapy, as previously concluded by the same analysis for ER+ and HER2+ BrCa (19, 20). Interestingly, RNA-Seq data from normal breast tissues and clinical samples of ER+ BrCa, HER2+ BrCa, and TNBC showed that the expression of CDK8, CDK19, and CCNC was the highest in TNBC (*SI Appendix*, Fig. S1*A*). Cancer Cell Line Encyclopedia (CCLE) data analysis showed that RNA expression of CDK8 (but not of CDK19 or CCNC) was significantly higher in TNBC cell lines than in cell lines from other subtypes of BrCa (*SI Appendix*, Fig. S1*B*). RNA and protein expression for these genes was positively correlated in the CCLE database, with the best correlation seen for CDK8 (*SI Appendix*, Fig. S1*C*).

### Effects of a CDK8/19 Inhibitor Alone and in Combination with mTOR and AKT Inhibitors on TNBC Cell Proliferation In Vitro.

**Fig. 2.**
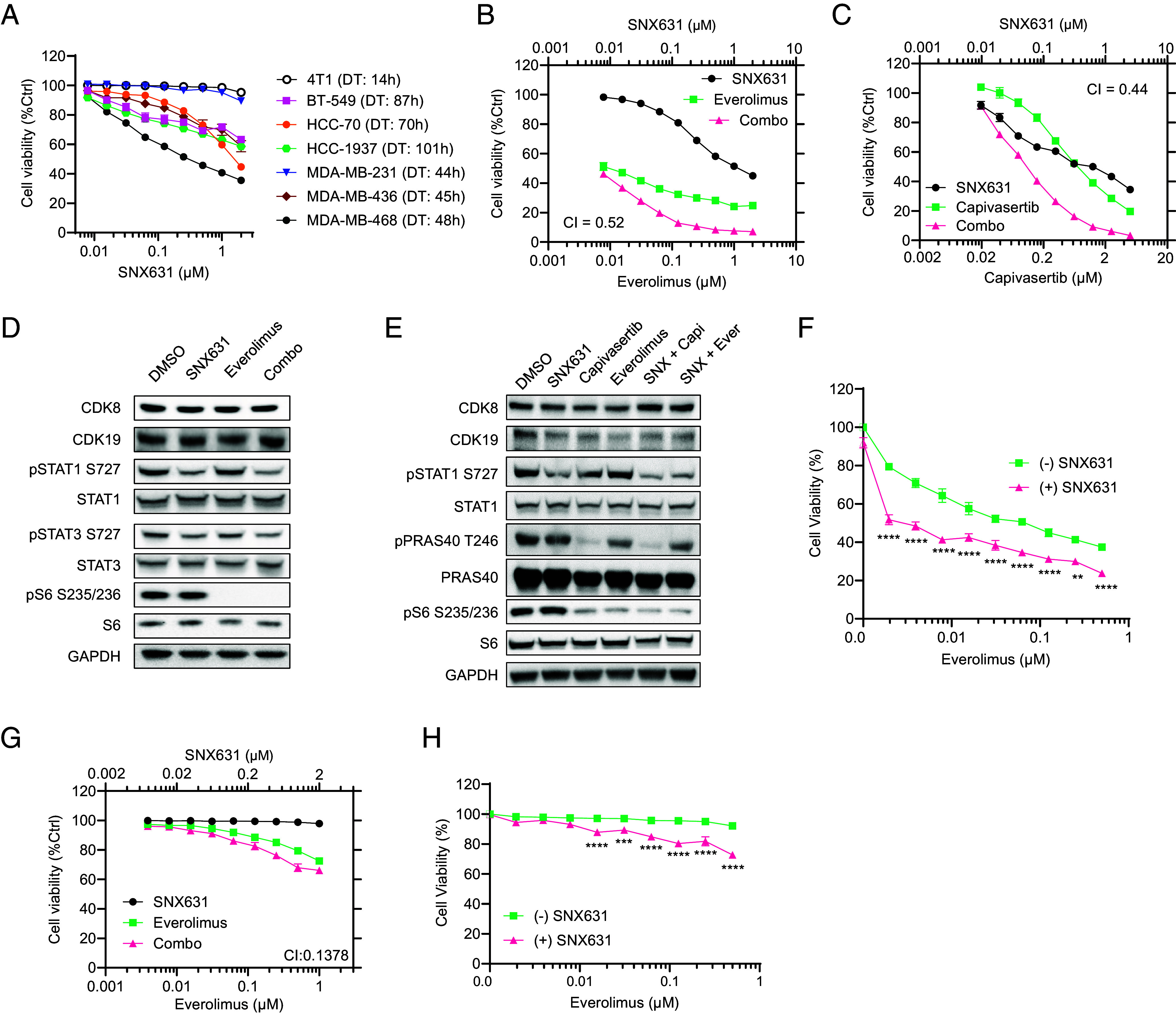
Effects of CDK8/19i SNX631 and its combinations with everolimus and capivasertib on TNBC cell lines in vitro. (*A*). Effects of different concentrations of SNX631 on the number of viable cells of the indicated TNBC cell lines after 7-d incubation, expressed as percent SRB measurements relative to untreated cells, mean ± SEM (n = 6). DT: doubling time. (*B* and *C*) Effects of different concentrations of SNX631, everolimus (*B*) or capivasertib (*C*) and their fixed-ratio combination with SNX631 on MDA-MB-468 cell number in a 7-d assay. Combination indices (CI) are shown. (*D* and *E*). Western blot analysis of the indicated proteins and their phosphorylated forms in MDA-MB-468 cells after 6-h (*D*) or 48-h (*E*) treatment with DMSO (control), 500 nm SNX631, 10 nM everolimus, 1 µM capivasertib, or their indicated combinations. (*F*). Effects of different concentrations of everolimus, with or without 500 nM SNX631, on BT549 cells in a 7-d assay. *P*-values: ****P < 0.0001; **P < 0.005 (*G*). Effects of different concentrations of SNX631, everolimus, and their fixed-ratio combination on MDA-MB-231 cells in a 7-d assay; CI value is shown. (*H*). Effects of different concentrations of everolimus, with or without 500 nM SNX631, on 4T1 cells in a 7-d assay. *P*-values: ****P < 0.0001; ***P < 0.0005.

To determine whether CDK8/19 kinase activity affects TNBC cell proliferation in vitro, we measured the effects of 7-day treatment with a selective CDK8/19i SNX631 (20) on the proliferation of six human and one murine TNBC cell lines. The responses of these cell lines to the CDK8/19i together with their doubling times are shown in Fig. 2*A*. While most of the human cell lines (MDA-MB-468, HCC-70, HCC-1937, MDA-MB-436, and BT-549) showed variable inhibition by SNX631, the compound had no effect in MDA-MB-231 and murine 4T1 cells.

Since SNX631-responsive MDA-MB-468 cells are PTEN-deficient and carry a PI3KCA mutation, we asked whether CDK8/19 inhibition would improve the response of these cells to mTOR and AKT inhibitors, by conducting synergy analysis of these inhibitors using the Combination Index (CI) method of Chou-Talalay (24), at fixed concentration ratios with SNX631. MDA-MB-468 cells were responsive to an mTOR (mTORC1) inhibitor everolimus and a pan-AKT inhibitor capivasertib. Combining these inhibitors with SNX631 led to a strong synergistic effect, as indicated by CI < 1 and the increase in the maximal cell growth inhibition relative to individual drugs (Fig. 2 *B* and *C*). CI analysis was validated by another experiment using arrays comprising different concentrations of SNX631 and everolimus and analyzing the data using SynergyFinder (25) that evaluates synergy based on four other mathematical and statistical models. All four models indicate statistically significant synergy scores for the drug combination (*SI Appendix*, Fig. S2).

We then asked whether the synergy of CDK8/19 and AKT/mTOR inhibitors could result from synergistic inhibition of the corresponding kinases. In one set of immunoblotting assays (Fig. 2*D*), MDA-MB-468 cells were treated with DMSO (control), SNX631 (500 nM), everolimus (10 nM), or their combination for 6 h. In the other set (Fig. 2*E*), cells were treated for 48 h using the same drugs as well as capivasertib (1 μM) and a combination of capivasertib and SNX631. Western blotting was carried out for CDK8, CDK19, and for both total and S727-phosphorylated STAT1 and STAT3; STAT phosphorylation is exerted directly (but not exclusively) by CDK8/19 (26, 27). Everolimus and capivasertib, by themselves or in combination with SNX631, had no apparent effect on CDK8 or CDK19 levels and did not potentiate the inhibition of STAT1 or STAT3 phosphorylation by SNX631 (Fig. 2 *D* and *E*). On the other hand, mTOR and AKT inhibitors suppressed the phosphorylation of ribosomal protein S6 at S235/236, and capivasertib inhibited the phosphorylation of the AKT target pPRAS40, but neither of these effects were noticeably affected by the addition of SNX631 (Fig. 2 *D* and *E*). Hence, the observed synergy between AKT/mTOR and CDK8/19 inhibitors does not appear to reflect synergistic inhibition of the corresponding kinases and is more likely to be driven by the transcriptional effects of Mediator kinases, as shown for other CDK8/19i drug combinations in vitro (11, 17–20).

Among the cell lines that showed in vitro response to the CDK8/19i alone (Fig. 2*A*), BT-549 cells are PTEN-deficient. We have investigated whether the addition of SNX631 at 500 nM concentration (which decreases BT-549 cell number by only 8.1% after 7 d) would affect BT-549 response to everolimus. As in the case of MDA-MB-468, the CDK8/19i increased the effect of everolimus at all tested drug concentrations (Fig. 2*F*).

We also tested the effects of everolimus and its combination with SNX631 in MDA-MB-231 and 4T1 TNBC cell lines that do not respond to the CDK8/19i (Fig. 2*A*) and have no known genetic aberrations in the PI3K/AKT/mTOR pathway. MDA-MB-231 cells showed a moderate response to everolimus that was synergistically increased in a fixed-ratio combination with SNX631 (Fig. 2*G*). On the other hand, 4T1 cells were totally unresponsive to everolimus but showed a moderate but significant response when everolimus was combined with 500 nM SNX631 (Fig. 2*H*), indicating an apparent synergy.

### Effects of CDK8/19 Inhibitors Alone and in Combination with mTOR and AKT Inhibitors on In Vivo growth of Cell Line-based and Patient-Derived Xenograft (PDX) TNBC Models.

**Fig. 3.**
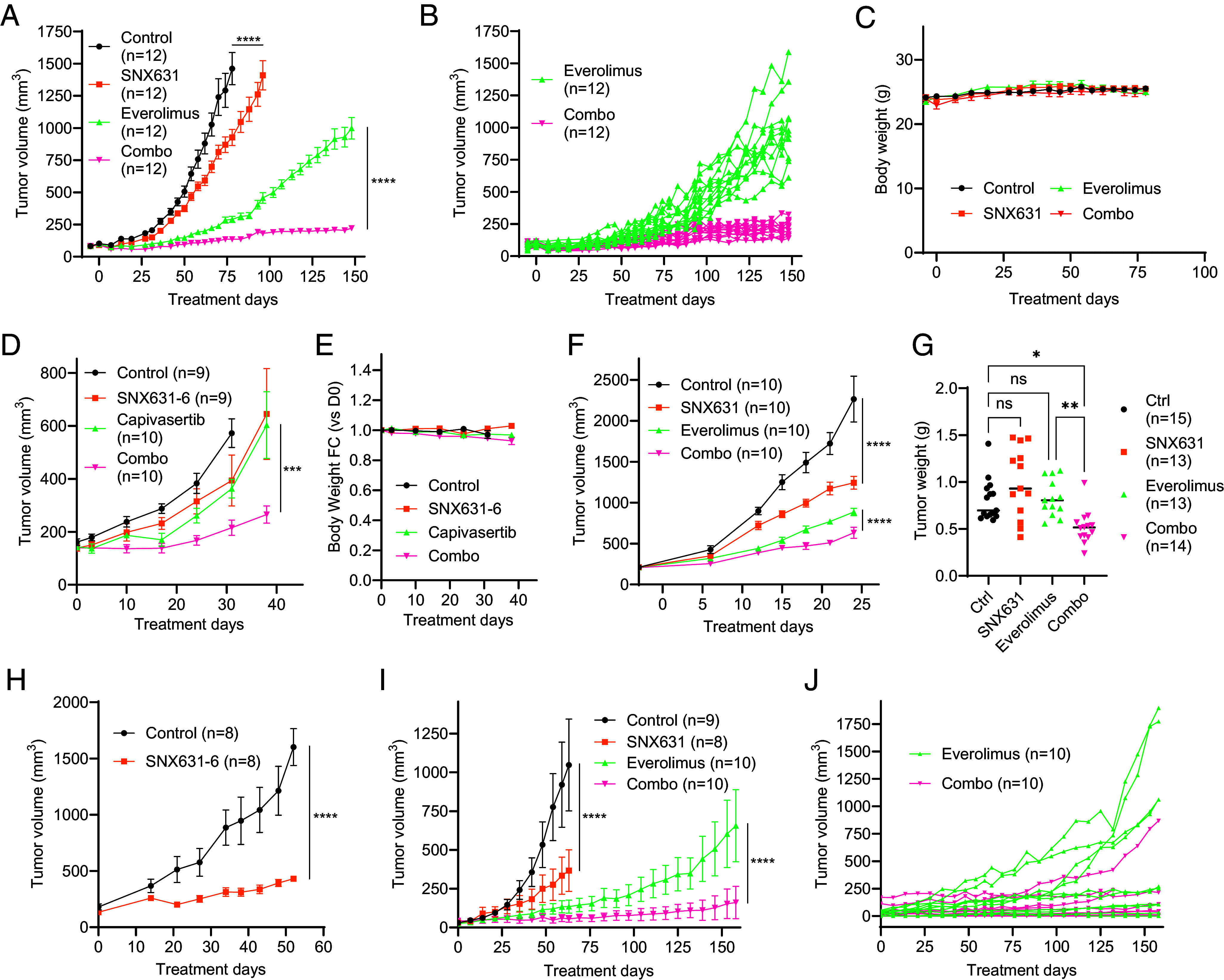
Effects of SNX631 or SNX631-6 and their combinations with everolimus or capivasertib on in vivo growth of different TNBC models. Group sizes (n) are shown in the corresponding labels. Mixed-effects ANOVA with Geisser-Greenhouse correction was performed to compare treatment group differences in all tumor growth comparisons. ****P < 0.0001; ***P < 0.001; **P < 0.01; *P < 0.05; ns, not significant. (*A*) Tumor growth curves of MDA-MB-468 xenografts in female NSG mice receiving vehicle controls, SNX631-medicated diet (350 ppm), everolimus (2 mg/kg, q.d.), or everolimus+SNX631 combination, mean ± SEM. (*B*) Individual tumor growth curves for the everolimus and combination arms of the study in (*A*). (*C*) Body weight changes for the animals in the study in (*A*), mean ±SEM. (*D*) Tumor growth curves of MDA-MB-468 xenografts receiving vehicle controls, SNX631-medicated diet (350 ppm), capivasertib (150 mg/kg, q.d.), or capivasertib+SNX631 combination, mean ± SEM. (*E*) Body weight changes for the animals in the study in (*D*); mean ± SEM. (*F*) Tumor growth curves of MDA-MB-231 xenografts receiving vehicle controls, SNX631-medicated diet (350 ppm), everolimus (3 mg/kg, q.d.), or everolimus+SNX631 combination, mean ±SEM. (*G*) Final tumor weights of 4T1 syngrafts after 14-d growth in female Balb/c receiving SNX631 (20 mg/kg, b.i.d.), everolimus (5 mg/kg, q.d.), or everolimus+SNX631 combination, evaluated by ordinary one-way ANOVA followed by Sidak’s multiple comparison test. (*H*) Tumor growth curves of PEN_175 PDX receiving vehicle control or SNX631-6-medicated diet (350 ppm), mean ± SEM. (*I*) Tumor growth curves of PEN_061 PDX receiving vehicle controls, SNX631-medicated diet (350 ppm), everolimus (3 mg/kg, q.d.), or everolimus+SNX631 combination, mean ±SEM. (*J*) Individual tumor growth curves for the everolimus and combination arms of the study in (*I*).

We analyzed the effects of SNX631, mTOR inhibitor everolimus, and their combination on in vivo growth of xenografts formed by MDA-MB-468 cells in female NSG mice. SNX631 alone showed a moderate but significant effect on MDA-MB-468 tumor growth (Fig. 3*A*). In contrast, everolimus alone completely suppressed MDA-MB-468 xenografts for ~50 d, but all the everolimus-treated tumors eventually started growing exponentially, indicating the adaptation to everolimus (Fig. 3*B*). However, when everolimus and SNX631 were combined, the combination-treated tumors did not resume their growth for the entire 150-d period of the study (Fig. 3 *A* and *B*). Everolimus and SNX631, individually and in combination, were very well tolerated as indicated by the lack of body weight loss (Fig. 3*C*) or other adverse reactions. Similarly, SNX631-6 [an equipotent analog of SNX631 (11)] potentiated the tumor-suppressive effect of the AKT inhibitor capivasertib in MDA-MB-468 xenografts, but since capivasertib did not fully suppress tumor growth, it was not possible to conclude whether the effect of SNX631-6 was due to the prevention of tumor adaptation to capivasertib (Fig. 3*D*). The capivasertib/SNX631-6 combination was also well tolerated (Fig. 3*E*).

We also tested the effects of SNX631, everolimus and their combination on MDA-MB-231 human TNBC xenografts. Although SNX631 had no effect on these cells in vitro (Fig. 2*A*), it produced significant tumor suppression in vivo (Fig. 3*F*). Everolimus had only a modest effect on MDA-MB-231 growth in vitro (Fig. 2*G*) but showed strong tumor growth inhibition in vivo, and the effect of everolimus was increased by the addition of SNX631 (Fig. 3*F*).

We also investigated the effects of SNX631, everolimus and their combination on the growth of 4T1 orthotopic syngrafts in immunocompetent Balb/c mice. In this case, the in vivo effects were concordant with the in vitro results (Fig. 2 *A* and *H*), with neither everolimus nor SNX631 showing a significant effect on the final tumor weights, but the drug combination significantly decreased the size of 4T1 tumors (Fig. 3*G*).

We further analyzed the effects of SNX631/SNX631-6 on the growth of two patient-derived xenograft (PDX) models of TNBC, PEN_175, and PEN_061 (28). SNX631-6 inhibited PEN_175 growth almost completely (Fig. 3*H*), whereas SNX631 significantly inhibited PEN_061 growth (Fig. 3*I*). We tested the effects of everolimus and everolimus/SNX631 combination in PEN_061 and obtained similar results to the MDA-MB-468 study: strong but temporary inhibition of PDX growth by everolimus alone, followed by apparent adaptation to everolimus that was counteracted by the CDK8/19i (Fig. 3*I*). Four of 10 PEN_061 tumors grew exponentially during the 158-d study after the initial suppression by everolimus, in contrast to just 1 of 10 combination-treated tumors (Fig. 3*J*).

The growth of PEN_175 and PEN_061 orthotopic PDXs was also inhibited by Senexin B, a less potent CDK8/19i which is structurally unrelated to SNX631 (19) (*SI Appendix*, Fig. S3 *A* and *B*). We also measured the effect of Senexin B on the number of circulating tumor cells (CTC) in mouse blood, an early indicator of metastatic spread, and found that the CTC number was strongly reduced by the CDK8/19i (*SI Appendix*, Fig. S3 *C* and *D*).

### Transcriptomic Mechanisms of the Prevention of In Vivo Adaptation to Everolimus by CDK8/19 Inhibition.

Since Mediator kinases function as transcriptional regulators, we have analyzed transcriptomic changes associated with the development of in vivo adaptation to everolimus and the effects of the CDK8/19i in MDA-MB-468 xenografts. In addition to the tumors collected in the long-term (LT) study in Fig. 3*A*, we also collected tumors from a separate short-term (ST) study, after 30 d of treatment with the vehicle and after 38 d of treatment with everolimus, when tumor growth was still suppressed by the mTOR inhibitor (*SI Appendix*, Fig. S4*A*). We then carried out RNA-Seq analysis of tumor samples from the ST and the LT studies, analyzing separately the data for the tumor (human) and stromal (mouse) RNA-Seq. Everolimus and SNX631 affected both tumor and stromal gene expression, as illustrated by volcano plots in *SI Appendix*, Fig. S4 *B* and *C*.

To identify tumor genes that could be involved in the in vivo adaptation to everolimus and the prevention of such adaptation by CDK8/19i, we selected differentially expressed genes (DEGs) affected by different treatments using a 1.5-fold change of median gene expression and false discovery rate (FDR) <0.05 as cutoffs for DEG selection. The resulting DEG lists were further filtered to remove the genes that differed between the control tumors in the ST and LT studies and could therefore be affected by differences in the tumor size. SNX631 alone affected the smallest number of DEGs (200, listed in Dataset S1), and 93% of them were upregulated. Remarkably, 61% of SNX631-upregulated DEGs were also upregulated by ST or LT everolimus treatments (*SI Appendix*, Fig. S4*D*), suggesting a similarity between the effects of CDK8/19 and mTOR inhibitors.

**Fig. 4.**
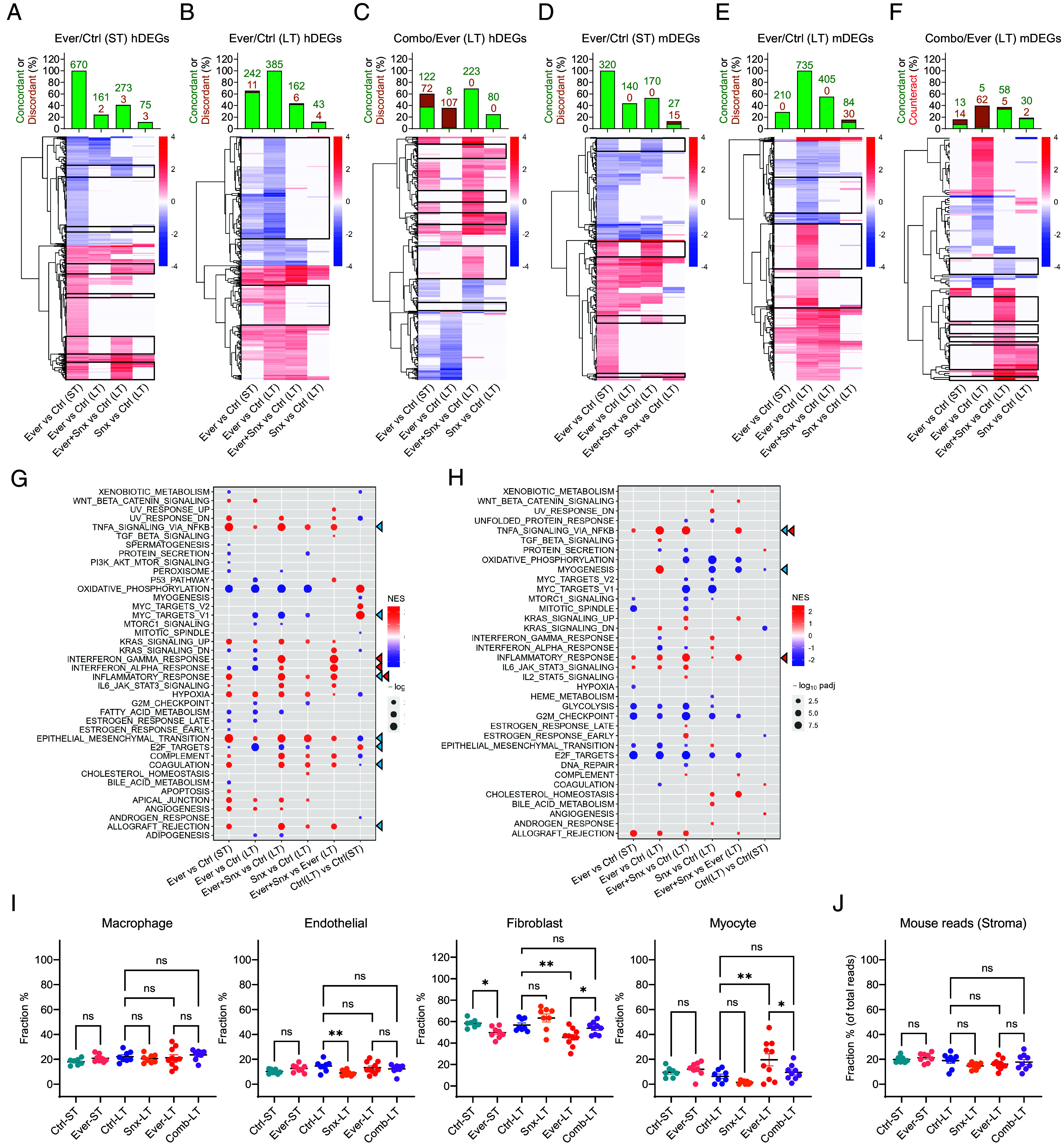
RNA-Seq analysis of the effects of everolimus, SNX631, and their combination in MDA-MB-468 xenografts. (*A*–*F*) Heatmaps of tumor (human) (*A*–*C*) and stromal (mouse) (*D*–*F*) DEGs affected by ST everolimus treatment relative to ST control (*A* and *D*), LT everolimus treatment relative to LT control (*B* and *E*) and combination treatment relative to LT everolimus (*C* and *F*) in the indicated comparisons. Bar diagrams on top of the heatmaps show the percent and numbers of the corresponding DEGs that show a statistically significant change (as defined by FDR<0.05) in the same direction (concordant) or the opposite direction (discordant) in the indicated comparisons. (*G* and *H*) Effects of ST and LT treatments with everolimus, SNX631 and their combination on the affected hallmark pathways for tumor (human) (*G*) and stromal (mouse) (*H*) RNA-Seq data. Blue triangles: pathways that show the most pronounced differences between ST and LT everolimus-treated tumors. Red triangles: pathways that are differentially affected by everolimus+SNX631 versus everolimus but are not similarly affected by SNX631 alone. (*I* and *J*) Fractions of the indicated stromal cell types (CIBERSORTx analysis) (*I*) and of all mouse reads (*J*) in the indicated tumor arms; *P*-values are shown for the indicated comparisons: **P < 0.01; *P < 0.05; ns, not significant.

Surprisingly, the number of tumor DEGs affected by everolimus in the ST study (670 DEGs, Dataset S2) was higher than in the LT study (385 DEGs, Dataset S3). While 63% of the DEGs affected by the LT everolimus treatment were similarly affected in the ST, only 24% of the genes affected by this drug in the ST were similarly affected in the LT (Fig. 4 *A* and *B*). Hence, everolimus adaptation that developed after LT treatment was associated with the abrogation of most of the drug effects found in the everolimus-responsive (ST) tumors. The addition of SNX631 to everolimus in the LT study restored some of the ST everolimus responses, increasing the number of concordantly affected DEGs from 161 to 273 (DEGs restored by the combination treatment are marked by boxes in Fig. 4*A*). On the other hand, the addition of the CDK8/19i decreased the number of LT everolimus DEGs from 385 to 162, as indicated by boxes in Fig. 4*B*. We have also analyzed DEGs that were differentially affected by everolimus+SNX631 combination relative to LT everolimus (323 DEGs, Dataset S4); such DEGs were most likely to include genes contributing to the prevention of everolimus adaptation. Most of these DEGs belonged to one of three categories: i) 107 affected by LT everolimus but not similarly affected by the drug combination, ii) 111 affected by the drug combination and by ST everolimus but not by LT everolimus treatment, and iii) 89 affected by the drug combination but not by everolimus alone (marked by boxes in Fig. 4*C*), including 63 DEGs that were also unaffected by SNX631 alone. Hence, the adaptive everolimus resistance is associated with partial loss of the transcriptomic response in tumor cells of the drug-responsive tumors. The addition of the CDK8/19i partially restores this response, counteracts many of the effects specific to the LT everolimus treatment, and elicits transcriptional changes unique to the drug combination.

The effects on the stromal genes had both similarities and differences with the effects found in tumor genes. While SNX631 alone upregulated almost all of the tumor genes, only 239 of 384 stromal DEGs affected by SNX631 alone (Dataset S5) were upregulated (*SI Appendix*, Fig. S4*C*). Then, 32% of SNX631-upregulated stromal genes were also upregulated by ST or LT everolimus, but almost all SNX631-downregulated stromal DEGs were either unaffected or affected in the opposite direction by LT everolimus treatment (*SI Appendix*, Fig. S4*E*). Hence, the similarity between the effects of mTOR and CDK8/19i inhibitors is limited to upregulated genes.

In contrast to the tumor DEGs where more genes were affected by everolimus in the ST than in the LT, the number of stromal DEGs affected by ST everolimus (320 DEGs, Dataset S6) was much lower than in the LT (735 DEGs, Dataset S7). Further, 140 of 320 stromal DEGs affected by ST everolimus were similarly affected by LT everolimus, and the addition of SNX631 to everolimus slightly increased this number (to 170, marked with boxes in Fig. 4*D*), while strongly decreasing the response of LT everolimus DEGs (from 735 to 405, marked with boxes in Fig. 4*E*). Among 121 stromal DEGs that were differentially affected by the drug combination relative to LT everolimus (Dataset S8), 62 were affected by LT everolimus but not by the drug combination, whereas only 11 DEGs were similarly affected by ST everolimus and the drug combination but not by LT everolimus, and 42 DEGs were affected by the drug combination but not by the ST or LT everolimus (marked with boxes in Fig. 4*F*), including 28 that were also unaffected by SNX631 alone. Hence, transcriptomic changes of stromal cells associated with the prevention of the everolimus adaptation by SNX631 differ quantitatively from those in tumor cells, with much less restoration of the ST response to everolimus, a greater counteraction of the LT everolimus response and a higher fraction of drug combination-specific effects.

Gene Set Enrichment Analysis (GSEA) of 50 hallmark pathways in tumor cells revealed the pathways that were differentially affected in different treatment arms (Fig. 4*G*). Among the pathways that show the most pronounced differences between ST and LT everolimus-treated tumors and may be related to everolimus adaptation (blue triangles), we note the TNFA signaling and EMT pathways that were induced to a greater degree by ST relative to LT everolimus, and proliferation-related E2F and MYC targets, which were inhibited by LT more than by ST everolimus treatment (despite the fact that everolimus-treated tumors were growing only in LT). Among the pathways that are differentially affected by everolimus+SNX631 versus everolimus but are not similarly affected by SNX631 alone (red triangles), the strongest changes were seen for the overlapping interferon (IFN)γ/IFNα/inflammatory response pathways that were selectively upregulated by the drug combination. The apoptosis pathway was increased in the everolimus-responsive ST-treated tumors but not in the drug-adapted LT-treated tumors, and it was not upregulated by the drug combination. Oxidative phosphorylation was the most strongly downregulated pathway by everolimus (both ST and LT) and by SNX631, but it was not differentially affected by the drug combination (Fig. 4*G*).

GSEA of stromal cells (Fig. 4*H*) showed that TNFA signaling and myogenesis pathways were upregulated to a greater degree by LT than by ST everolimus. TNFA signaling and the related inflammatory response pathway were further upregulated but the myogenesis pathway was downregulated by the addition of SNX631. Surprisingly, the effects of everolimus (a mTORC1 inhibitor) on PI3K_AKT_mTOR and mTORC1 signaling pathways were insignificant or minor in both tumor and stromal cells, and these pathways were unaffected by SNX631. Changes in stromal gene expression could reflect treatment effects on the transcriptomes of specific stromal cell types or changes in the distribution of different cell types in the tumor-infiltrating stroma. To address the latter possibility, we used CIBERSORTx (29) to estimate the abundances of different cell types in the tumor-associated stromal cell population. This analysis showed that the macrophage fraction was not significantly affected by any treatments, and the endothelial cell fraction was decreased by SNX631 but not by everolimus. Both ST and LT everolimus treatments decreased the fibroblast fraction, and this effect was reversed by the combination of everolimus and SNX631. The fraction of cells with myocyte characteristics was increased by LT everolimus treatment, with the drug combination producing a similar value to the control (Fig. 4*I*), in parallel with the effects observed for the myogenesis pathway in GSEA(Fig. 4*H*). The differences in stromal cell type abundances were not due to differential representation of mouse RNA in different treatment arms (Fig. 4*J*).

RNA-Seq analysis identified multiple tumor and stromal genes, changes of which could have contributed mechanistically to tumor suppression by the drug combination. *SI Appendix*, Fig. S5 shows the expression of individual tumor and stromal genes that presented the biggest differences between the LT everolimus and combination treatments and that were not strongly affected by SNX631 alone; possibly pertinent functions of these genes are summarized in *SI Appendix*, Table S1 (tumor genes) and *SI Appendix*, Table S2 (stromal genes). Among the tumor genes (*SI Appendix*, Fig. S5*A*), we note some that have been implicated in mTOR activation (MAP2K6 and IL17RC) or inhibition (GJB3) and were upregulated or downregulated, respectively, in everolimus-adapted (LT) tumors. We also note the proapoptotic protein PMAIP1 (NOXA) that was downregulated in the drug-adapted tumors, and transcription factors NFIX, EGR1, and HES2 implicated in tumor promotion or suppression, which were differentially affected by LT everolimus and the drug combination. Among the stromal genes (*SI Appendix*, Fig. S5*B*), we note that the everolimus+SNX631 combination upregulated an angiogenesis inhibitor Tnmd and putative tumor-suppressive proteins Acaca, Per3, and Ccl11, whereas Aldh1a2, a tumor stimulating stromal factor, was downregulated by the combination. Changes in these genes could contribute to stroma-mediated suppression of tumors treated with the drug combination.

### CDK8/19 Inhibition Extends the Survival of Pulmonary Metastatic Disease in 4T1 Murine TNBC Model.

Although SNX631 did not affect in vitro proliferation (Fig. 2*A*) or in vivo primary tumor growth of murine 4T1 cells (Fig. 3*H*), CDK8/19i were previously shown to selectively affect the metastatic over the primary tumors in colon cancer (12). We therefore used the highly metastatic 4T1 model to test the effects of Mediator kinase inhibition in a spontaneous metastasis assay. In this assay, 4T1 cells were injected orthotopically in the mammary fat pad of female Balb/c mice. Fourteen days after injection, the presence of lung metastases was tested by explanting the dissociated lung tissues in media containing 60 µM 6-thioguanine (6-TG), to which 4T1 cells are resistant. All the lung explants of mice carrying 4T1 orthotopic tumors gave rise to multiple colonies of 6-TG resistant tumor cells (*SI Appendix*, Fig. S6*A*), indicating that all the lungs already contained metastatic cells by 14 d after tumor implantation. For the metastasis survival studies, primary tumors were surgically removed 15 to 17 d after inoculation, by which time all the mice carried metastases in the lungs, to which they eventually succumbed (representative macroscopic images of lung tumors at the terminal point are shown in *SI Appendix*, Fig. S6*B*).

**Fig. 5.**
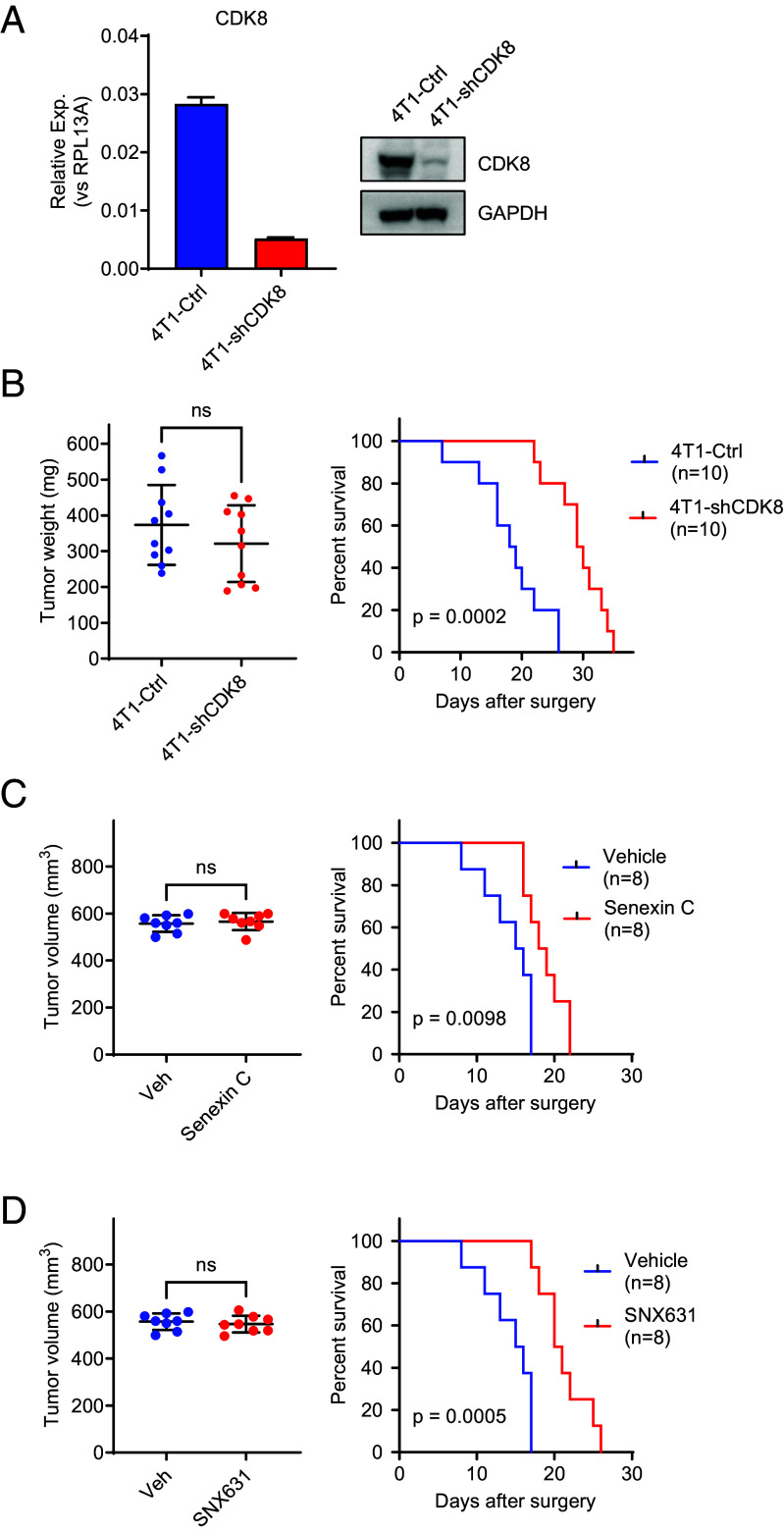
Effects of CDK8/19 inhibition on the survival of 4T1 TNBC metastatic disease. (*A*) Depletion of Cdk8 mRNA (QPCR assay, *Left*) and protein (western blot, *Right*) in 4T1-shCdk8 derivative relative to vector-transduced 4T1 control cells. (*B*) *Left*: 4T1 control and 4T1-shCdk8 primary tumor weights at resection (d 17), n = 10. *Right*: KM survival plot of mice bearing 4T1 control and 4T1-shCdk8 tumors after primary tumor resection. (*C*) *Left*: 4T1 tumor weights at resection (d 15) for groups assigned to control and Senexin C treatment arms, n = 8. *Right*: KM survival plot after primary 4T1 tumor resection in mice receiving vehicle control or Senexin C (40 mg/kg, b.i.d.). (*D*) *Left*: 4T1 tumor weights at resection (d 15) for groups assigned to control and SNX631 treatment arms, n = 8. *Right*: KM survival plot after primary 4T1 tumor resection in mice receiving vehicle control or SNX631 (25 mg/kg, b.i.d.).

In the first study, we generated a 4T1 derivative with shRNA-mediated knockdown of Cdk8 [which is expressed in 4T1 at a higher level than Cdk19 according to RNA-Seq data in (30)]. Cdk8 knockdown was carried out as previously described (12) and was nearly complete according to RNA and protein analysis (Fig. 5*A*). The resulting shCdk8 derivative and control 4T1 cells (transduced with an insert-free vector) were used for orthotopic injection, and primary tumors were excised 17 d after inoculation. Although there was no significant difference in the sizes of the resected primary tumors between shCdk8 and control arms (Fig. 5*B*, *Left*), Cdk8 knockdown was associated with a pronounced increase in the host survival in the spontaneous metastasis assay (1.6-fold extension of median survival, *P* = 0.0002) (Fig. 5*B*, *Right*).

The effect of shCdk8 in the spontaneous metastasis model could be due to the inhibition of the metastatic spread or to the effect on metastatic tumors after they were established. To test whether Cdk8/19 inhibition affects the survival of already established pulmonary metastases, we have investigated the survival effects of systemic CDK8/19i treatment that was started after the resection of the primary tumors, using two structurally unrelated CDK8/19i, Senexin C (31), and SNX631. Primary tumors were removed 15 d after injection, when tumor sizes reached 500 to 600 mm^3^. After the resection, mice were randomized into two groups by the primary tumor size (Fig. 5 *C* and *D*, *Left*) and were then treated by oral gavage with Senexin C (40 mg/kg, b.i.d.), SNX631 (25 mg/kg, b.i.d.), or the corresponding vehicle controls. Treatment with either CDK8/19i significantly increased mouse survival of the metastatic disease (Fig. 5 *C* and *D*, *Right*), with SNX631 exerting a stronger effect in agreement with its greater potency. Hence, Mediator kinase inhibition extends the survival of already established TNBC lung metastases, even when it has no effect on the primary tumor size.

## Discussion

CDK8/19i are unique among anticancer drugs, since they rarely inhibit the proliferation of tumor cells but more often suppress the adaptation of such cells to environmental challenges, such as metastatic growth or therapy. The present study reveals that these effects of CDK8/19i are especially pronounced in the context of TNBC, a major unmet medical need. The response of TNBC cell lines to CDK8/19i in vitro was highly heterogeneous, with two cell lines (MDA-MB-231 and 4T1) showing no response. When tested in vivo, however, MDA-MB-231 xenografts responded to CDK8/19i therapy, as did MDA-MB-468 xenografts (that were responsive in vitro) and both tested TNBC PDXs, one of which (PEN_175) was the most responsive among all in vivo models. We previously showed the in vivo specificity of tumor suppression by CDK8/19i in other tumor types (11, 19, 20). Hence, the clinical niche for the CDK8/19i is not limited to tumors that respond to Mediator kinase inhibition in vitro.

Although the primary tumor growth of murine 4T1 cells was not significantly affected by CDK8/19i, Cdk8 knockdown in tumor cells and SNX631 treatment of mice carrying established lung metastases extended the survival of the metastatic disease in this model. The latter finding parallels the preferential effect of CDK8/19i against hepatic metastases versus primary tumors in colon cancer (12) and the results with the murine E0771 TNBC model, where Cdk8 knockdown decreased the metastatic burden in the lungs but not the primary tumor growth (22). Although the effect of CDK8/19i on the metastatic burden was not directly measured in our study, the observed significant extension of metastatic disease survival found in the 4T1 model, coupled with prior results, indicates the potential of the CDK8/19i for the treatment of TNBC lung metastases, a major cause of lethality in this disease.

The strongest effects of the CDK8/19i were observed in drug combinations, in agreement with bioinformatic analysis that revealed that the survival correlations of CDK8/CDK19/CCNC expression in TNBC become much stronger when the analysis is limited to patients who received therapy after the sample collection. Here, we have investigated the effects of CDK8/19i combined with mTOR inhibitor everolimus or AKT inhibitor capivasertib. Remarkably, CDK8/19i not only showed in vitro synergy with everolimus and capivasertib in TNBC models but also strikingly increased their in vivo activity. For mouse models used in this study, this was especially apparent for the everolimus combination, which fully suppressed the growth of all tumors (in the case of MDA-MB-468) or all but one tumor (in the case of PEN_061 PDX) for at least 150 d, indicating the prevention of in vivo adaptation to everolimus. The combinations of the CDK8/19i with everolimus and capivasertib were very well tolerated in mice, with no significant weight loss.

Transcriptomic analysis of tumors that remained responsive to everolimus (ST treatment) or became adapted to the drug (LT treatment) revealed that much of the ST transcriptional response was lost in the tumor cells after the LT treatment, likely contributing to the adaptive drug resistance. In contrast, stromal cells of the same tumors showed more changes after LT everolimus treatment, indicating continued stromal effects of the drug. The addition of the CDK8/19i to everolimus in the LT study restored some of the ST drug response of tumor cells, including upregulation of the TNFA pathway and prevented changes in gene expression that developed in the LT treated tumors and could contribute to the adaptive everolimus resistance, such as upregulation of p38 activator MAP2K6 or downregulation of the proapoptotic gene PMAIP1. In addition, combining mTOR and CDK8/19 inhibitors elicited transcriptional changes that were specific to the drug combination, such as upregulation of the IFNα/γ pathways in tumor cells and of extracellular proteins associated with tumor suppression (e.g., Lrp1b) or inhibition of angiogenesis (Tnmd) in stromal cells. Hence, prevention of in vivo adaptation to everolimus by the CDK8/19i is associated with effects on the expression of multiple tumor and stromal genes that regulate tumor growth and drug response.

The effects of the CDK8/19i and everolimus on stromal proteins implicated in tumor growth could contribute to in vivo suppression of tumor models (such as 4T1 and MDA-MB-231) that were not responsive in vitro. We note that some of the transcriptomic changes in the stroma could be due to changes in its cell type composition, as revealed by CIBERSORTx analysis. This analysis suggested an increase in tumor-associated myocytes that occurred in everolimus-adapted tumors and was counteracted by CDK8/19i, which could reflect changes in tumor invasion of the muscle layer, which was previously shown to be inhibited by CDK8/19i (32).

In summary, our results warrant the exploration of CDK8/19i for the treatment of TNBC, both as single agents, with activity against the metastatic disease, and as combinations with approved drugs, in particular mTOR and AKT inhibitors. The PI3K/AKT/mTOR pathway was estimated to be upregulated in up to 70% of all breast cancers, including TNBC (3) but inhibitors of this pathway have been approved so far only for ER+ breast cancer and have not shown clinical efficacy in TNBC. The results of the present study suggest that the addition of CDK8/19i to mTOR and AKT inhibitors could potentially transform the utility of such drugs for TNBC therapy.

## Materials and Methods

All mouse studies were approved by the University of South Carolina and West Virginia University Institutional Animal Care and Use Committees. Mouse tumor studies, cell culture, shRNA knockdown, western blotting, transcriptomics, statistical, and bioinformatic analyses were done largely as previously described (8, 11, 12, 20). Additional details are provided in *SI Appendix*.

## Supplementary Material

Appendix 01 (PDF)

Dataset S01 (XLSX)

Dataset S02 (XLSX)

Dataset S03 (XLSX)

Dataset S04 (XLSX)

Dataset S05 (XLSX)

Dataset S06 (XLSX)

Dataset S07 (XLSX)

Dataset S08 (XLSX)

## Data Availability

RNA-Seq data have been deposited in GEO (GSE271325) (33). All study data are included in the article and/or supporting information.
