# Supplementary material for: Mediator kinase inhibitors suppress triple-negative breast cancer growth and extend tumor suppression by mTOR and AKT inhibitors
Source: Proc Natl Acad Sci U S A. 2024 Nov 14;121(47):e2414501121. doi: 10.1073/pnas.2414501121 (PMC11588072; doi:10.1073/pnas.2414501121)
Supplement: Supplementary file 1 — Appendix 01 (PDF) [file pnas.2414501121.sapp.pdf]

SUPPLEMENTARY INFORMATION

- Supplementary Materials and Methods
- Supplementary Figure Legends
- Supplementary Figures S1-S5
- Supplementary Tables S1-S3
- Supplementary Information References
- Separate Excel files: Datasets S1-S8

Supplementary Materials and Methods

Cell culture and chemicals. All cell lines were obtained from ATCC except where noted. Cell lines were regularly tested for mycoplasma by MycoAlert Plus mycoplasma detection kit (Lonza, Morrisville, NC). 4T1, HCC1937 and HCC70 cells were maintained in RPMI-1640 media (ThermoFisher Scientific, Waltham, MA) supplemented with 10% (or 20% for HCC70) fetal bovine serum (FBS; Cytiva, Marlborough, MA), 1% penicillin/streptomycin, and 2 mM glutamine. BT549, MDA-MB-231, MDA-MB-436 and MDA-MB-468 cells were maintained in DMEM high glucose media (ThermoFisher Scientific) supplemented with 10% FBS (or 10% fetal clone II serum for MDA-MB-231), 1% penicillin/streptomycin, 2 mM glutamine and (for MDA-MB-436 and MDA-MB-468) with 1 x non-essential amino acids. 4T1 derivatives expressing murine Cdk8 shRNA or control lentiviral vector were generated by lentiviral transduction using previously described vectors, shRNA sequence targeting CDK8 (GTCTTATCAGTGGGTTGATTC) and procedures for lentiviral transduction, QPCR and western blotting (1).

CDK8/19i SNX631, SNX631-6, Senexin B and Senexin C were synthesized for Senex Biotechnology (Columbia, SC). Everolimus and capivasertib were purchased from MedChemExpress (Monmouth Junction, NJ).

Cell proliferation assays. Cells were seeded into 96-well plates (1000-5000 cells/well, depending on doubling time). After 24 h, cells were treated with SNX631, everolimus, capivasertib or their combinations at the indicated concentrations. Viable cells were measured by sulforhodamine B (SRB) assay. Synergy of drug combinations was assessed by Combination Index (CI) values, calculated using CompuSyn software. For SynergyFinder analysis, MDA-MB-468 cells were seeded in triplicate across three 96-well plates, with each plate comprising 60 wells of cells (6 rows by 10 columns) treated with combinations of different concentrations of SNX631 (from 0 to

1,000 nM) and everolimus (from 0 to 500 nM) over 7 days. The results of the SRB assay (% of untreated control) were analyzed through the SynergyFinder+ web application (2).

Western blot. ~4 x 10^5 cells were plated in 6-well plates and allowed ~24 h to attach prior to treatments. Treated cells were then lysed in RIPA buffer with protease and phosphatase inhibitors. Whole cell lysates were resolved on 4-12% ExpressPlus PAGE gel (#M41215, GenScript Biotech, Piscataway, NJ), transferred to PVDF membranes, blocked with 5% non-fat milk and incubated with the following primary antibodies overnight at 4°C: STAT1 (#sc592, 1:500; Santa Cruz Biotechnologies, Dallas, TX; or #9172S, 1:1000; Cell Signaling Technology, Danvers, MA), STAT3 (#9139S, 1:1000; Cell Signaling Technology), phospho-STAT1 Ser727 (#8826S, 1:1000; Cell Signaling Technology), phospho-STAT3 Ser727 (#9134S, 1:1000; Cell Signaling Technology), GAPDH (#2118S, 1:1000, Cell Signaling Technology), S6 (#2217S, 1:1000, Cell Signaling Technology), phospho-S6 Ser235/236 (#2211S, 1:1000, Cell Signaling Technology), PRAS40 (#2691, 1:1000, Cell Signaling Technology), phospho-PRAS40 (Thr246) (#2997S, 1:1000, Cell Signaling Technology). Membranes were then washed with TBST (Tris-buffered saline with 0.1% Tween 20) and blotted with HRP-conjugated anti-Mouse (NXA931, GE healthcare) or anti-Rabbit (NA934, GE Healthcare, Chicago, IL) secondary antibodies. Protein bands were detected with Western Lighting Plus ECL reagent (NEL105001EA, Perkin Elmer) in ChemiDoc TouchTM (Bio-Rad Laboratories, Hercules, CA) machine.

Primary tumor model *in vivo* studies. All mouse studies were approved by the University of South Carolina (USC) and West Virginia University (WVU) Institutional Animal Care and Use Committees (IACUC). Female NOD.Cg-PrkdcscidIL2rgtm1Wjl/SzJ (NSG) and Balb/c mice (aged 6 weeks) were obtained from The Jackson Laboratory (Bar Harbor, ME) and allowed to habituate for 7-10 days before experiments. Tumor volumes were measured with calipers. Once tumors reached the endpoint, mice were euthanized, and tumors were removed, cut into pieces and preserved for RNA analysis (in RNAlater) and for histology (in 10% formalin), where indicated.

For implantation, 2x10^6 MDA-MB-468 or MDA-MB-231 cells were suspended in PBS, mixed 1:1 with 50% Matrigel (Corning, Oneonta, NY) and injected into NSG mice orthotopically under fat pad. Once tumors reached ~200 mm^3 (for MDA-MB-231) or ~80 mm^3 (for MDA-MB-468), mice were randomized into groups with similar-size tumors for further treatments. For the MDA-MB-468 short term everolimus study, mice received vehicle (70% polyethylene glycol (PEG) 400/30% propylene glycol (PG)) or 2 mg/kg everolimus in the same vehicle, q.d.. For the MDA-MB-468 long term everolimus combination study, mice receiving SNX631 were put on 350 ppm SNX631-medicated diet (350 ppm) four days prior to the start of 2 mg/kg, q.d everolimus or vehicle

treatment, to accommodate to food flavor. For the MDA-MB-468 capivasertib combination study, mice received capivasertib in the same vehicle at 100 mg/kg q.d. or vehicle alone, with or without 350 ppm SNX631-6 medicated diet. For the MDA-MB-231 everolimus combination study, 350 ppm SNX631 medicated diet was started 3 days prior to 3 mg/kg, q.d. everolimus treatment.

For the 4T1 everolimus combination study, 1 x 10^5 cells were suspended in Matrigel and injected into Balb/c mice under fat pad. Once tumor volumes reached ~100 mm^3, mice were randomized into 4 treatment groups that received oral gavage with vehicle (70% PEG 400/30% PG), 20 mg/kg SNX631 (b.i.d. by oral gavage in the same vehicle) and 5 mg/kg everolimus, q.d.

PEN_175 and PEN_061 PDX models were described in (3). For the studies at WVU, tumor tissues were implanted into the mammary fat of female NSG mice. Treatment was started when tumors reached ~100-150 mm^3. Mice were randomly assigned to groups that received control vehicle (1% Dextrose, 6.25% 2-Hydroxypropyl-β-cyclodextrin) or Senexin B (dimaleate salt, in the same vehicle) by oral gavage at 75 mg/kg b.i.d. for the first 4 weeks and then at 100 mg/kg q.d.; treatment was continued until at least one tumor in the control group reached 1,000 mm^3. Terminal blood samples were collected via cardiac puncture. Red blood cells were lysed using RBC lysis Buffer (Myltenyi Biotec); the samples were co-stained for human-specific CD298 and DAPI and analyzed by flow cytometry using fluorescently conjugated antibodies (anti-CD298-PE) or isotype controls (Myltenyi Biotec, cat#130-126-377). Circulating tumor cells (CTC) were defined as DAPI+/CD298+ cells.

For the studies at USC, PDX tissues were implanted subcutaneously to the right flank of female NSG mice. Once tumors reached ~50-100 mm^3, mice were randomized into treatment groups. For the PEN_175 study, two groups of mice received SNX631-6 medicated food (350 ppm) or control diet. In the PEN_061 study, four groups received control diet and vehicle, 3 mg/kg everolimus q.d., 350 ppm SNX631 medicated diet or the combination of SNX631 and everolimus, with SNX631 given for 9 days prior to the start of everolimus treatment, for mice to accommodate to food flavor.

<u>4T1 spontaneous metastasis studies.</u> 5 × 10^5 4T1 control or shCdk8 cells in 100 µl DMEM were injected into the second mammary gland of 7-week-old female BALB/c mice. The presence of metastases in the lungs was evaluated 14 days after tumor inoculation. The single-lobed left lungs were dissected out from euthanized animals and dissociated with a collagenase type I/IV cocktail at 37℃ for two hours. After dissociation, singlet cell suspensions were seeded in P150 plates in DMEM medium with 10% FBS and 60 µM 6-thioguanine. Ten days later, colonies were stained

by crystal violet and counted. For the metastasis survival studies, the primary tumors were surgically removed and weighed 15 or 17 days after inoculation. Mice were then carefully monitored without treatment (for the comparison of shCdk8 and control derivatives) or treated with Senexin C (40 mg/kg in in 5% dextrose), b.i.d., SNX631 (25 mg/kg in in 0.5% sodium carboxylmethyl cellulose), b.i.d., or the corresponding vehicle controls. Mice were euthanized when moribund; the presence of metastases was confirmed by necropsy. Mouse survival in different arms was compared by Kaplan-Meier (KM) plot analysis.

Statistical analysis.

For comparing endpoint values among groups numbering more than two, ordinary one-way ANOVA followed by Sidak's multiple comparison test was utilized. For comparisons between two treatment groups, a two-tailed unpaired t-test was employed. Multiple comparisons, whether parametric or nonparametric, were conducted using the two-stage linear step-up procedure of Benjamini, Krieger, and Yekutieli. Differences between treatment groups over time were evaluated using mixed-effects ANOVA with Geisser-Greenhouse correction. In KM survival analysis, survival curves were compared using the Gehan-Breslow-Wilcoxon test. All statistical analyses were performed with GraphPad Prism 10 software.

Bioinformatic analysis.

Survival correlations of CDK8/CDK19/CCNC gene expression were analyzed using KMplotter gene chip data for breast cancer (4). The analysis was carried out for all cancers defined as ER-negative and HER2-negative based on array data, or after the exclusion of systemically untreated patients. Analysis was conducted using JetSet best probe sets and by auto-selecting the best cutoff.

RNA-Seq data from clinical breast cancer patient samples were retrieved from TCGA database (BRCA-TCGA project, https://portal.gdc.cancer.gov/projects/TCGA-BRCA) and cBioportal database (https://www.cbioportal.org/study/summary?id=brca_mbcproject_2022) using the R packages TCGAbiolinks and cBioPortalData. A total of 113 normal breast tissues, 443 ER+ breast cancers, 37 HER2+ breast cancers, and 116 TNBC samples were included in the analysis. Log2-transformed FPKM values and pseudo-number (log2(FPKM + 1)) were used for expression analysis. Gene expression was plotted with the ggplot2 package. RNA expression levels of CDK8, CDK19, and CCNC across 63 BrCa cell lines (30 non-TNBC and 33 TNBC) were obtained from the curated RNA-Seq data of cell models in the Cancer Cell Line Encyclopedia (CCLE)

database via DepMap website. Protein expression levels of CDK8, CDK19, and CCNC across 30 BrCa cell lines were sourced from the normalized protein quantitation data available at CCLE website.

Transcriptomic analysis. Tumor tissues preserved in RNAlater were mechanically homogenized and RNA was extracted using miRNeasy Mini Kit (QIAGEN) according to the manufacturer's protocol. Library preparation, next generation sequencing and post-processing of raw data were performed by the Functional Genomics Core (FGC) of the USC Center for Targeted Therapeutics, as described (5). Differential expression (DE) analysis was performed in R, using the DESeq2 pipeline. The normalized count data were fitted to a negative binomial distribution model using a generalized linear model (GLM) framework, and the Benjamini-Hochberg procedure was used to control the false discovery rate (FDR) for multiple testing. The log2 fold change (logFC) was calculated by computing the base-2 logarithm of the ratio of the median normalized expression values (transcripts per million, TPM) for each group. A small pseudocount was added to the expression values of all genes before calculating the logFC to avoid division by zero. The logFC and FDR values calculated from the DESeq2 pipeline were used to select differentially expressed genes (DEGs), with the selection criteria of FC > 1.5 and FDR < 0.05. Genes differentially expressed between the ST and LT control groups (FC > 1.2 and FDR < 0.05) were excluded for the heatmap analysis to compare the effects of drug treatments between the two studies. For the genes where DE analysis did not reach statistical significance (FDR >= 0.05), logFC values were set to zero. In the Gene set enrichment analysis (GSEA) was conducted using the fgsea package with specific gene sets downloaded from the Human and Mouse Molecular Signatures Database (MSigDB). The cell type abundance in stroma was estimated using the analytical tool CYBERSORTx (https://cibersortx.stanford.edu/) with the mouse gene expression profile and the HNSCC_Signature_matrix provided from the website. Detailed information about the individual RNA-Seq samples (sample title and description, GEO accession number) is listed in Table S3.

Supplementary Figure Legends

Figure S1. CDK8/CDK19/CCNC expression in clinical samples and cell lines of different BrCa subtypes.

(A) RNA expression of CDK8, CDK19 and CCNC in normal breast (n = 113), ER+ BrCa (n = 443), HER2+ BrCa (n = 37) and TNBC (n = 116) (data from TCGA and cBioPortal). Asterisks mark p-values: ****<0.0001; **<0.01, ns, not significant.

(B) RNA expression of CDK8, CDK19 and CCNC in cell lines representing TNBC (n=33) versus other subtypes of BrCa (n =30) (data from CCLE).

(C) Correlations for RNA and protein expression of CDK8, CDK19 and CCNC in BrCa cell lines (data from CCLE, n= 30). Slope and Pearson correlation coefficients (r) were calculated by linear regression and correlation analysis.

Figure S2. SynergyFinder evaluation of synergy between everolimus and SNX631 in MDA-MB-468 cells.

Evaluation of synergy according to the Highest Single Agent (HSA), Loewe additivity (LOEWE), Bliss independence (BLISS), and Zero Interaction Potency (ZIP) models.

Figure S3. Effects of Senexin B on TNBC PDX models.

(A) Tumor growth curves of PEN_175 in female NSG mice receiving vehicle control (n=3) or Senexin B (75 mg/kg, b.i.d. for the first 4 weeks, followed by 100 mg/kg, b.i.d.) (n=4), mean ±SEM. **(B)** Tumor growth curves of PEN_061 PDX in female NSG mice receiving vehicle control (n=2) or Senexin B (n=3), mean ±SEM. **(C,D)** Circulating tumor cells (CTC) counts in terminal blood samples of mice bearing PEN_175 (**C**) and PEN_061 (**D**) PDX from the studies in (A,B). p-values: ****<0.0001; **<0.01; *<0.05.

Figure S4. RNA-Seq analysis of the effects of everolimus and SNX631 in MDA-MB-468 xenografts.

(**A**) Tumor growth curves (mean ±SEM) of MDA-MB-468 xenografts in the short-term study in female NSG mice receiving vehicle control or SNX631 (in medicated food, 350 ppm). p-values: ****< 0.0001. (**B,C**) Volcano plots of RNA-Seq data for the indicated comparisons for the human (**B**) and mouse (**C**) genes. Differentially expressed genes (DEGs) passing the selection criteria (FC >1.5, FDR <0.05) are marked with red (upregulated) and blue (downregulated) dots. (**D,E**). Heatmaps of the human (**D**) and mouse (**E**) DEGs affected by SNX631 treatment. Bar diagrams on top of the heatmaps show the percent and the numbers of the corresponding DEGs that show a statistically significant change (as defined by FDR<0.05) in the same direction (concordant) or the opposite direction (discordant) in the indicated comparisons.

Figure S5. Expression of representative tumor (A) and stromal (B) genes in MDA-MB-468 xenografts under the indicated conditions.

Expression (log2[TPM+1]) of representative tumor **(A)** and stromal **(B)** genes in MDA-MB-468 xenografts under the indicated conditions (ST-Ctrl: n=7; ST-Ever: n=8; LT-Ctrl: n=8; LT-Snx: n=8; LT-Ever: n=10; LT-Combo: n=9).

Figure S6. Lung metastases in 4T1 model.

(A) Evaluation of 4T1 lung metastases based on *in vitro* growth of 6-TG resistant colonies from lungs of 5 female Balb/c mice 14 days after orthotopic implantation of 4T1 cells. (**B**) Representative macroscopic images of lungs from two animals at the endpoint.

Fig. S1

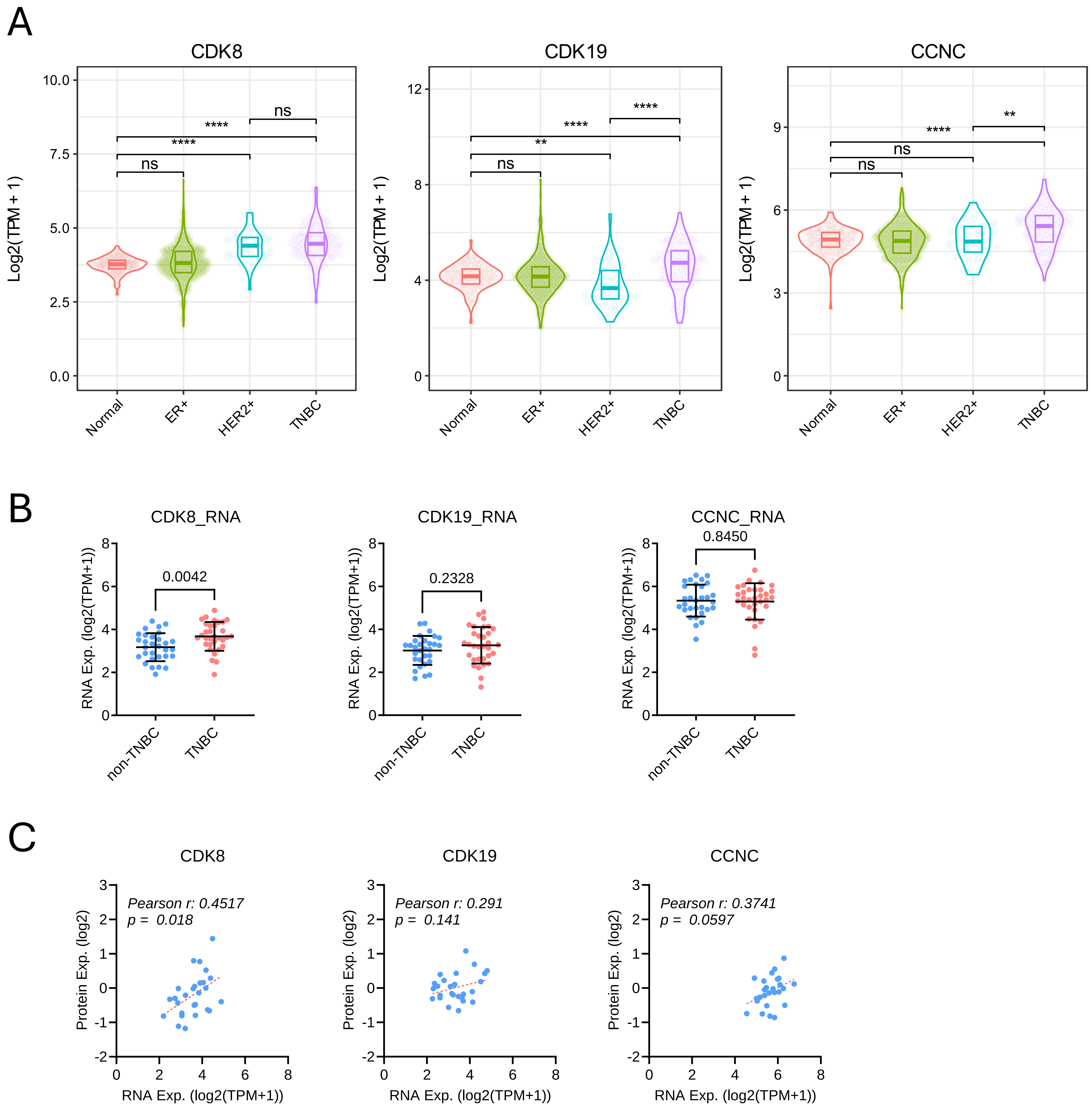
A
CDK8
CDK19
CCNC
Log2(TPM + 1)
Normal
ER+
HER2+
TNBC
ns
****
**
B
CDK8_RNA
CDK19_RNA
CCNC_RNA
RNA Exp. (log2(TPM+1))
0.0042
0.2328
0.8450
non-TNBC
TNBC
C
CDK8
CDK19
CCNC
Protein Exp. (log2)
RNA Exp. (log2(TPM+1))
Pearson r: 0.4517
p = 0.018
Pearson r: 0.291
p = 0.141
Pearson r: 0.3741
p = 0.0597

Fig. S2

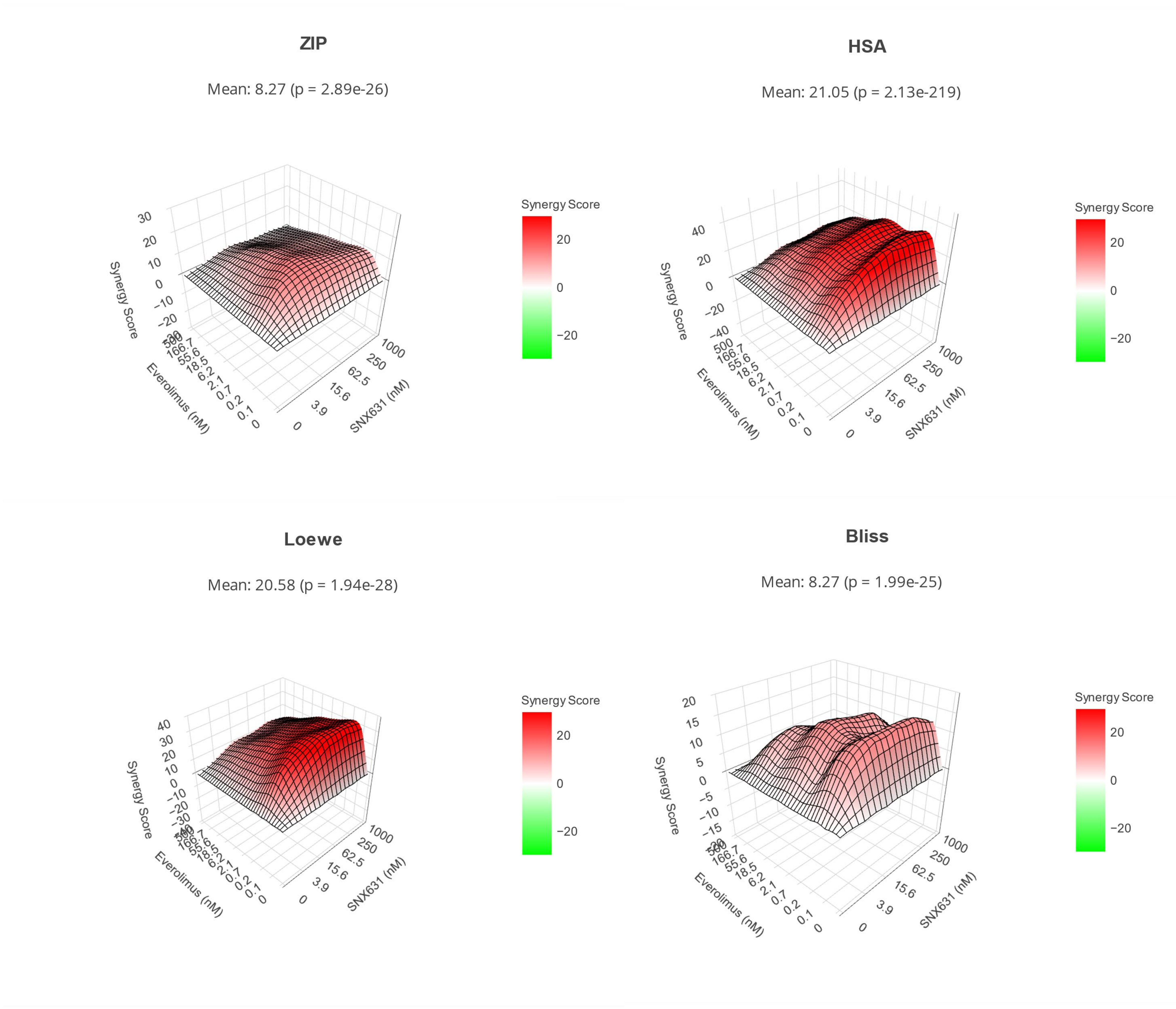
ZIP
Mean: 8.27 (p = 2.89e-26)
Synergy Score
Everolimus (nM)
SNX631 (nM)
HSA
Mean: 21.05 (p = 2.13e-219)
Synergy Score
Everolimus (nM)
SNX631 (nM)
Loewe
Mean: 20.58 (p = 1.94e-28)
Synergy Score
Everolimus (nM)
SNX631 (nM)
Bliss
Mean: 8.27 (p = 1.99e-25)
Synergy Score
Everolimus (nM)
SNX631 (nM)

Fig. S3

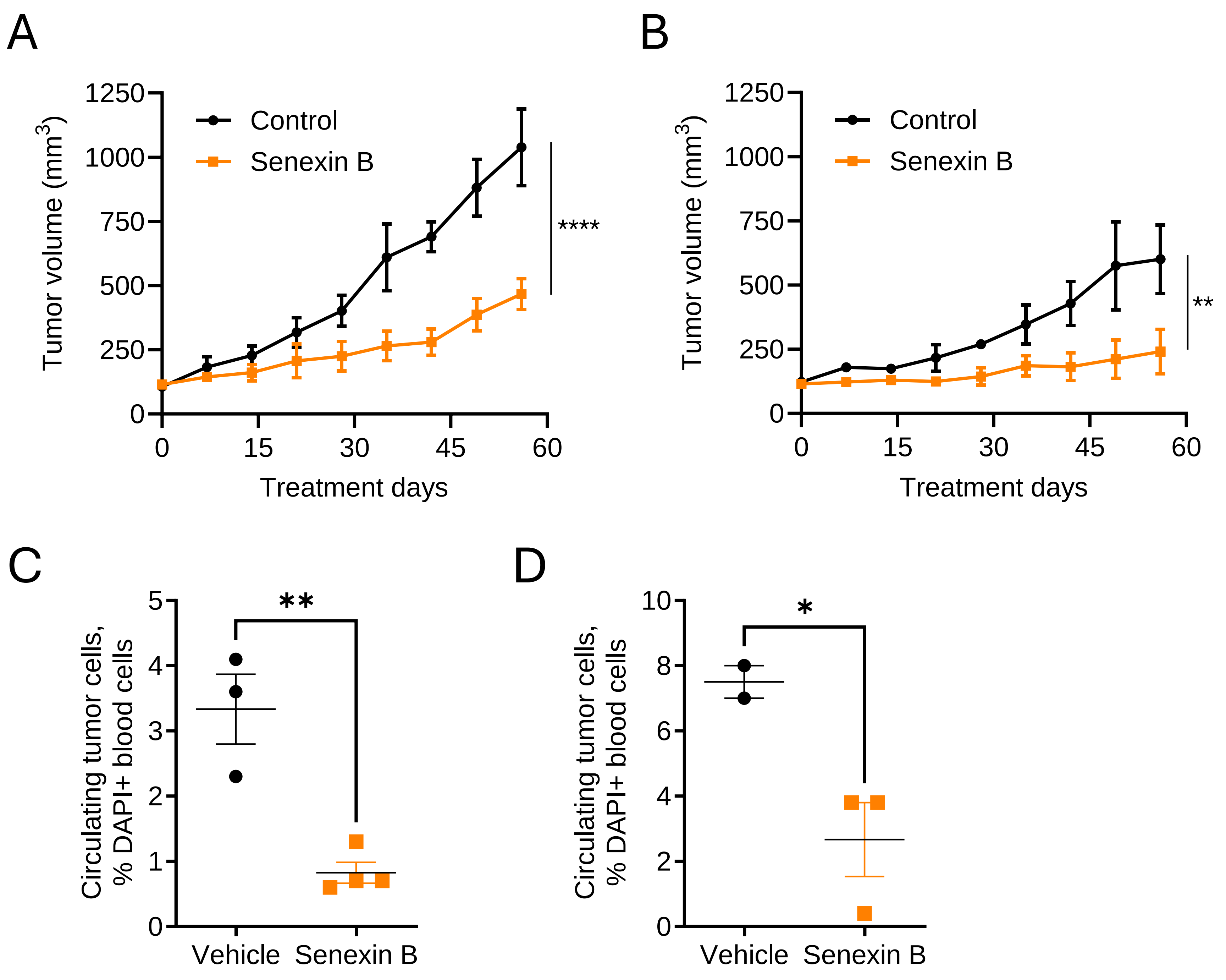

A
Tumor volume (mm^3)
1250
1000
750
500
250
0
Control
Senexin B
****
0
15
30
45
60
Treatment days
B
Tumor volume (mm^3)
1250
1000
750
500
250
0
Control
Senexin B
**
0
15
30
45
60
Treatment days
C
Circulating tumor cells,
% DAPI+ blood cells
5
4
3
2
1
0
**
Vehicle
Senexin B
D
Circulating tumor cells,
% DAPI+ blood cells
10
8
6
4
2
0
*
Vehicle
Senexin B

Fig. S4

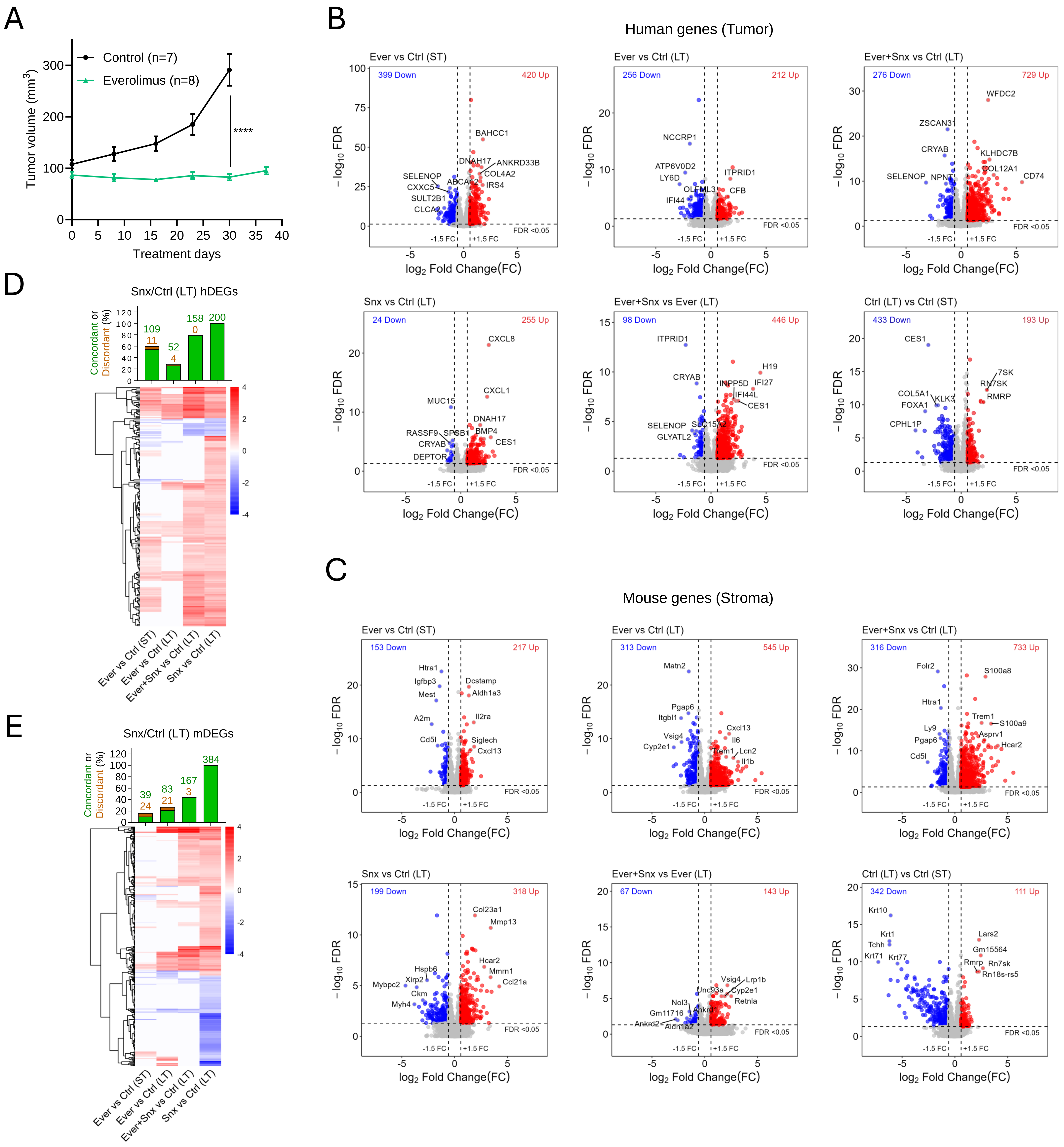
A
Control (n=7)
Everolimus (n=8)
Tumor volume (mm³)
Treatment days
****
B
Human genes (Tumor)
Ever vs Ctrl (ST)
399 Down
420 Up
Ever vs Ctrl (LT)
256 Down
212 Up
Ever+Snx vs Ctrl (LT)
276 Down
729 Up
Snx vs Ctrl (LT)
24 Down
255 Up
Ever+Snx vs Ever (LT)
98 Down
446 Up
Ctrl (LT) vs Ctrl (ST)
433 Down
193 Up
BAHCC1
DNAH17
ANKRD33B
COL4A2
IRS4
SELENOP
CXXC5
ABCA12
SULT2B1
CLCA2
NCCRP1
ATP6V0D2
LY6D
ITPRID1
OLFML3
CFB
IFI44
WFDC2
ZSCAN31
CRYAB
KLHDC7B
COL12A1
CD74
NPNT
CXCL8
CXCL1
MUC15
DNAH17
RASSF9
SPSB1
BMP4
CES1
DEPTOR
ITPRID1
H19
INPP5D
IFI27
IFI44L
SLC15A2
GLYATL2
CES1
7SK
RN7SK
RMRP
COL5A1
KLK3
FOXA1
CPHL1P
FDR <0.05
-1.5 FC
+1.5 FC
− log10 FDR
log2 Fold Change(FC)
C
Mouse genes (Stroma)
Ever vs Ctrl (ST)
153 Down
217 Up
Ever vs Ctrl (LT)
313 Down
545 Up
Ever+Snx vs Ctrl (LT)
316 Down
733 Up
Snx vs Ctrl (LT)
199 Down
318 Up
Ever+Snx vs Ever (LT)
67 Down
143 Up
Ctrl (LT) vs Ctrl (ST)
342 Down
111 Up
Htra1
Igfbp3
Mest
Dcstamp
Aldh1a3
A2m
Il2ra
Cd5l
Siglech
Cxcl13
Matn2
Pgap6
Itgbl1
Cxcl13
Il6
Vsig4
Cyp2e1
Trem1
Lcn2
Il1b
Folr2
S100a8
Trem1
S100a9
Ly9
Pgap6
Asprv1
Hcar2
Col23a1
Mmp13
Hspb6
Hcar2
Mmrn1
Xirp2
Ccl21a
Mybpc2
Ckm
Myh4
Vsig4
Lrp1b
Unc93a
Cyp2e1
Nol3
Retnla
Gm11716
Ankrd1
Ankrd2
Aldh1a2
Krt10
Krt1
Lars2
Tchh
Gm15564
Krt71
Krt77
Rmrp
Rn7sk
Rn18s-rs5
D
Snx/Ctrl (LT) hDEGs
Concordant or Discordant (%)
109
11
52
4
158
0
200
Ever vs Ctrl (ST)
Ever vs Ctrl (LT)
Ever+Snx vs Ctrl (LT)
Snx vs Ctrl (LT)
E
Snx/Ctrl (LT) mDEGs
Concordant or Discordant (%)
39
24
83
21
167
3
384
Ever vs Ctrl (ST)
Ever vs Ctrl (LT)
Ever+Snx vs Ctrl (LT)
Snx vs Ctrl (LT)

Fig. S5

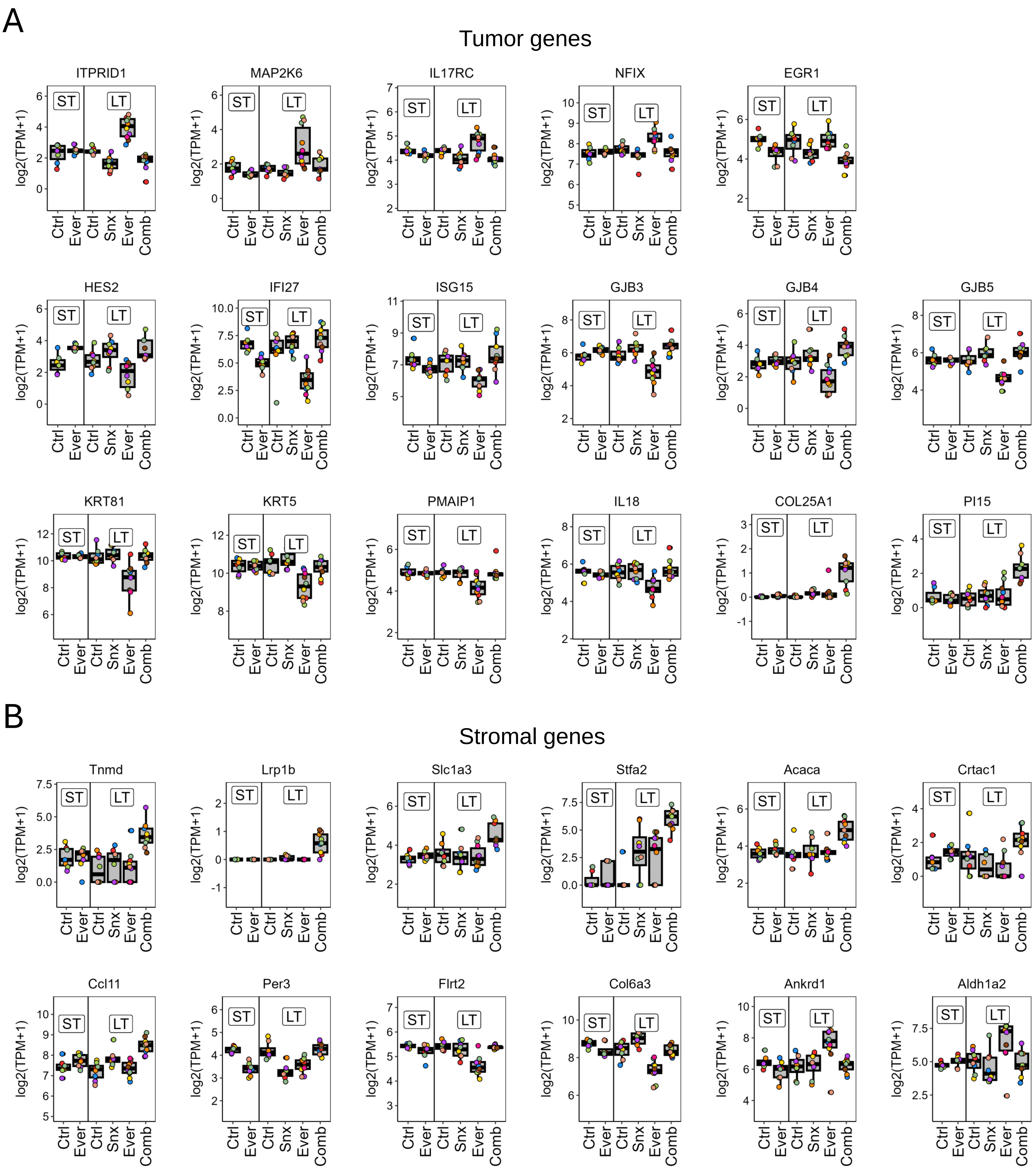
A
Tumor genes
ITPRID1
MAP2K6
IL17RC
NFIX
EGR1
HES2
IFI27
ISG15
GJB3
GJB4
GJB5
KRT81
KRT5
PMAIP1
IL18
COL25A1
PI15
B
Stromal genes
Tnmd
Lrp1b
Slc1a3
Stfa2
Acaca
Crtac1
Ccl11
Per3
Flrt2
Col6a3
Ankrd1
Aldh1a2
ST
LT
log2(TPM+1)
Ctrl
Ever
Ctrl
Snx
Ever
Comb

Fig. S6

A

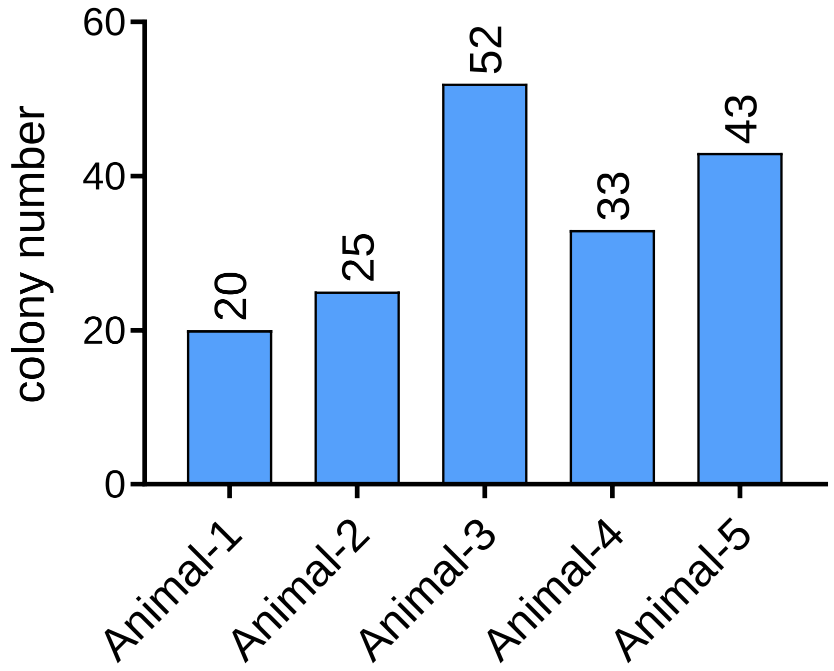

60
40
20
0
colony number
20
25
52
33
43
Animal-1
Animal-2
Animal-3
Animal-4
Animal-5

B

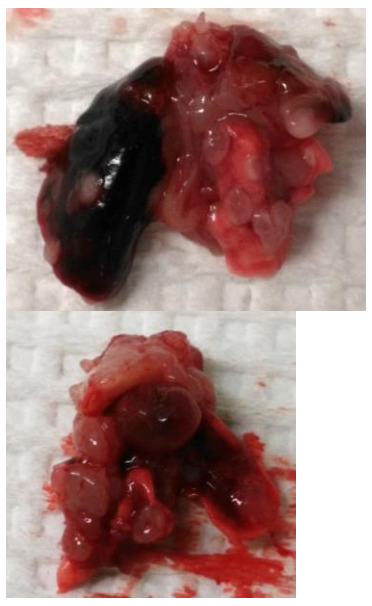

Table S1. Selected differentially expressed tumor genes (see Fig. S5A)

Gene	Full gene name	Pertinent function	Ref
ITPRID1 (CCDC129)	ITPR interacting domain containing 1	Sperm-specific antigen 2-related protein family. One of top 10 upregulated genes in lung adenocarcinomas.	(6)
MAP2K6 (MEK6)	Mitogen-activated protein kinase kinase 6	Essential component of p38 MAP kinase mediated signal transduction pathway. Mediates Akt-independent mTOR activation and rapamycin resistance.	(7)
IL17RC	Interleukin 17 receptor C	Involved in mTOR activation in injured epithelium.	(8)
NFIX	Nuclear factor I X	A member of the NFI family of transcription factors, altered in tumors, often promoting pro-tumorigenic functions. However, some studies suggest that NFIX can also have a tumor suppressor role.	(9)
EGR1	Early growth response factor 1	Transcription factor involved in tissue injury, immune responses, and fibrosis. EGR1 may participate in tumor cell proliferation, invasion, and metastasis and in tumor angiogenesis.	(10)
HES2	Hes family bHLH transcription factor 2	Potential tumor suppressor in neuroblastoma.	(11)
IFI27, ISG15	Interferon alpha inducible protein 27, ISG15 ubiquitin like modifier	Interferon-stimulated genes, high expression correlated with complete remission in patients with cervical cancer treated with cisplatin.	(12)
GJB3, GJB4, GJB5	Gap junction proteins beta 3-5	Members of beta-type connexin family, co-regulated in lung adenocarcinoma. GJB3 may inhibit the PI3K/AKT/mTOR pathway.	(13)
KRT81, KRT5	Keratins 81 and 5	Basal-like cytokeratins, differentially expressed in TNBC subtypes.	(14)
PMAIP1 (NOXA)	Phorbol-12-myristate-13-acetate-induced protein 1	Pro-apoptotic Bcl-2 family protein that belongs to a subclass of BH3-only proteins.	(15)
IL18	Interleukin-18	A member of IL-1 superfamily of cytokines, with pro- and anti-cancerous effects.	(16)
COL25A1	Collagen type XXV alpha 1 chain	High expression associated with longer relapse-free survival in breast cancers	KMplot.com
PI15	Peptidase inhibitor 15	High expression associated with longer relapse-free survival in breast cancers	KMplot.com

Table S2. Selected differentially expressed stromal genes (see Fig. S5B).

Gene	Full gene name	Pertinent function	Ref
Tnmd	Tenomodulin	Angiogenesis inhibitor	(17)
Lrp1B	Low density lipoprotein-related protein 1B	Tumor suppressor expressed in tumor and stroma in ovarian cancer	(18)
Slc1a3	Solute carrier family 1 (glial high affinity glutamate transporter), member 3	SLC1A3 overexpression activates the PI3K/AKT pathway	(19)
Stfa2	Stefin A2	Competitive inhibitor of intracellular papain-like cysteine proteases	(20)
Acaca	Acetyl-Coenzyme A carboxylase alpha	The downregulation of ACACA in lung fibroblasts triggered a senescent and inflammatory phenotypic shift of lung fibroblasts. Knock-in of ACACA prevented lung metastasis in the MMTV-PyVT mouse model.	(21)
Crtac1	Cartilage acidic protein 1	CRTAC1 enhances the chemosensitivity of non-small cell lung cancer to cisplatin by eliciting RyR-mediated calcium release and inhibiting Akt1 expression.	(22)
Ccl11	C-C motif chemokine ligand 11	CCL11-eosinophil axis suppresses pancreatic cancer progression	(23)
Per3	Period circadian clock 3	PER3 plays anticancer roles in the oncogenesis and progression of breast cancer via regulating MEK/ERK signaling pathway.	(24)
Flrt2	Fibronectin leucine rich transmembrane protein 2	FLRT2 is expressed preferentially in abnormalized vessels of advanced colorectal cancers in humans.	(25)
Col6a3	Collagen, type VI, alpha 3	COL6A3 is highly presented on tumor stroma across multiple solid cancers due to a tumor-specific alternative splicing event.	(26)
Ankrd1	Ankyrin repeat domain 1	ANKRD1 has been identified as an anti-inflammatory factor and is related to tumor drug resistance.	(27)
Aldh1a2	Aldehyde dehydrogenase 1 family member A2	Tumor-stimulating factor in glioblastoma-associated macrophages	(28)

Table S3. List of RNA-Seq samples in GEO database.

RNASeq Sample ID	Sample Title	GEO Series ID	GEO Sample ID	Sample Description
#001	MDA-MB-468_Ctrl_ST_Rep1	GSE271325	GSM8374547	MDA-MB-468 xenograft growing in female NSG, treated with vehicle for 30 days
#002	MDA-MB-468_Ctrl_ST_Rep2	GSE271325	GSM8374548	MDA-MB-468 xenograft growing in female NSG, treated with vehicle for 30 days
#003	MDA-MB-468_Ctrl_ST_Rep3	GSE271325	GSM8374549	MDA-MB-468 xenograft growing in female NSG, treated with vehicle for 30 days
#004	MDA-MB-468_Ctrl_ST_Rep4	GSE271325	GSM8374550	MDA-MB-468 xenograft growing in female NSG, treated with vehicle for 30 days
#005	MDA-MB-468_Ctrl_ST_Rep5	GSE271325	GSM8374551	MDA-MB-468 xenograft growing in female NSG, treated with vehicle for 30 days
#006	MDA-MB-468_Ctrl_ST_Rep6	GSE271325	GSM8374552	MDA-MB-468 xenograft growing in female NSG, treated with vehicle for 30 days
#007	MDA-MB-468_Ctrl_ST_Rep7	GSE271325	GSM8374553	MDA-MB-468 xenograft growing in female NSG, treated with vehicle for 30 days
#008	MDA-MB-468_Everolimus_ST_Rep1	GSE271325	GSM8374554	MDA-MB-468 xenograft growing in female NSG, treated with everolimus (2mg/kg q.d.) for 38 days
#009	MDA-MB-468_Everolimus_ST_Rep2	GSE271325	GSM8374555	MDA-MB-468 xenograft growing in female NSG, treated with everolimus (2mg/kg q.d.) for 38 days
#010	MDA-MB-468_Everolimus_ST_Rep3	GSE271325	GSM8374556	MDA-MB-468 xenograft growing in female NSG, treated with everolimus (2mg/kg q.d.) for 38 days
#011	MDA-MB-468_Everolimus_ST_Rep4	GSE271325	GSM8374557	MDA-MB-468 xenograft growing in female NSG, treated with everolimus (2mg/kg q.d.) for 38 days
#012	MDA-MB-468_Everolimus_ST_Rep5	GSE271325	GSM8374558	MDA-MB-468 xenograft growing in female NSG, treated with everolimus (2mg/kg q.d.) for 38 days
#013	MDA-MB-468_Everolimus_ST_Rep6	GSE271325	GSM8374559	MDA-MB-468 xenograft growing in female NSG, treated with everolimus (2mg/kg q.d.) for 38 days
#014	MDA-MB-468_Everolimus_ST_Rep7	GSE271325	GSM8374560	MDA-MB-468 xenograft growing in female NSG, treated with everolimus (2mg/kg q.d.) for 38 days
#015	MDA-MB-468_Everolimus_ST_Rep8	GSE271325	GSM8374561	MDA-MB-468 xenograft growing in female NSG, treated with everolimus (2mg/kg q.d.) for 38 days
#016	MDA-MB-468_Ctrl_LT_Rep1	GSE271325	GSM8374562	MDA-MB-468 xenograft growing in female NSG, treated with vehicle for 78 days
#017	MDA-MB-468_Ctrl_LT_Rep2	GSE271325	GSM8374563	MDA-MB-468 xenograft growing in female NSG, treated with vehicle for 78 days
#018	MDA-MB-468_Ctrl_LT_Rep3	GSE271325	GSM8374564	MDA-MB-468 xenograft growing in female NSG, treated with vehicle for 78 days
#019	MDA-MB-468_Ctrl_LT_Rep4	GSE271325	GSM8374565	MDA-MB-468 xenograft growing in female NSG, treated with vehicle for 78 days
#020	MDA-MB-468_Ctrl_LT_Rep5	GSE271325	GSM8374566	MDA-MB-468 xenograft growing in female NSG, treated with vehicle for 78 days
#021	MDA-MB-468_Ctrl_LT_Rep6	GSE271325	GSM8374567	MDA-MB-468 xenograft growing in female NSG, treated with vehicle for 78 days
#022	MDA-MB-468_Ctrl_LT_Rep7	GSE271325	GSM8374568	MDA-MB-468 xenograft growing in female NSG, treated with vehicle for 78 days
#023	MDA-MB-468_Ctrl_LT_Rep8	GSE271325	GSM8374569	MDA-MB-468 xenograft growing in female NSG, treated with vehicle for 78 days
#024	MDA-MB-468_SNX631_LT_Rep1	GSE271325	GSM8374570	MDA-MB-468 xenograft growing in female NSG, treated with SNX631 (350ppm chow) for 96 days
#025	MDA-MB-468_SNX631_LT_Rep2	GSE271325	GSM8374571	MDA-MB-468 xenograft growing in female NSG, treated with SNX631 (350ppm chow) for 96 days
#026	MDA-MB-468_SNX631_LT_Rep3	GSE271325	GSM8374572	MDA-MB-468 xenograft growing in female NSG, treated with SNX631 (350ppm chow) for 96 days
#027	MDA-MB-468_SNX631_LT_Rep4	GSE271325	GSM8374573	MDA-MB-468 xenograft growing in female NSG, treated with SNX631 (350ppm chow) for 96 days
#028	MDA-MB-468_SNX631_LT_Rep5	GSE271325	GSM8374574	MDA-MB-468 xenograft growing in female NSG, treated with SNX631 (350ppm chow) for 96 days
#029	MDA-MB-468_SNX631_LT_Rep6	GSE271325	GSM8374575	MDA-MB-468 xenograft growing in female NSG, treated with SNX631 (350ppm chow) for 96 days
#030	MDA-MB-468_SNX631_LT_Rep7	GSE271325	GSM8374576	MDA-MB-468 xenograft growing in female NSG, treated with SNX631 (350ppm chow) for 96 days
#031	MDA-MB-468_SNX631_LT_Rep8	GSE271325	GSM8374577	MDA-MB-468 xenograft growing in female NSG, treated with SNX631 (350ppm chow) for 96 days
#032	MDA-MB-468_Everolimus_LT_Rep1	GSE271325	GSM8374578	MDA-MB-468 xenograft growing in female NSG, treated with everolimus (2mg/kg q.d.) for 148 days

#033	MDA-MB-468_Everolimus_LT_Rep2	GSE271325	GSM8374579	MDA-MB-468 xenograft growing in female NSG, treated with everolimus (2mg/kg q.d.) for 148 days
#034	MDA-MB-468_Everolimus_LT_Rep3	GSE271325	GSM8374580	MDA-MB-468 xenograft growing in female NSG, treated with everolimus (2mg/kg q.d.) for 148 days
#035	MDA-MB-468_Everolimus_LT_Rep4	GSE271325	GSM8374581	MDA-MB-468 xenograft growing in female NSG, treated with everolimus (2mg/kg q.d.) for 148 days
#036	MDA-MB-468_Everolimus_LT_Rep5	GSE271325	GSM8374582	MDA-MB-468 xenograft growing in female NSG, treated with everolimus (2mg/kg q.d.) for 148 days
#037	MDA-MB-468_Everolimus_LT_Rep6	GSE271325	GSM8374583	MDA-MB-468 xenograft growing in female NSG, treated with everolimus (2mg/kg q.d.) for 148 days
#038	MDA-MB-468_Everolimus_LT_Rep7	GSE271325	GSM8374584	MDA-MB-468 xenograft growing in female NSG, treated with everolimus (2mg/kg q.d.) for 148 days
#039	MDA-MB-468_Everolimus_LT_Rep8	GSE271325	GSM8374585	MDA-MB-468 xenograft growing in female NSG, treated with everolimus (2mg/kg q.d.) for 148 days
#040	MDA-MB-468_Everolimus_LT_Rep9	GSE271325	GSM8374586	MDA-MB-468 xenograft growing in female NSG, treated with everolimus (2mg/kg q.d.) for 148 days
#041	MDA-MB-468_Everolimus_LT_Rep10	GSE271325	GSM8374587	MDA-MB-468 xenograft growing in female NSG, treated with everolimus (2mg/kg q.d.) for 148 days
#042	MDA-MB-468_Combination_LT_Rep1	GSE271325	GSM8374588	MDA-MB-468 xenograft growing in female NSG, treated with everolimus+SNX631 combination for 148 days
#043	MDA-MB-468_Combination_LT_Rep2	GSE271325	GSM8374589	MDA-MB-468 xenograft growing in female NSG, treated with everolimus+SNX631 combination for 148 days
#044	MDA-MB-468_Combination_LT_Rep3	GSE271325	GSM8374590	MDA-MB-468 xenograft growing in female NSG, treated with everolimus+SNX631 combination for 148 days
#045	MDA-MB-468_Combination_LT_Rep4	GSE271325	GSM8374591	MDA-MB-468 xenograft growing in female NSG, treated with everolimus+SNX631 combination for 148 days
#046	MDA-MB-468_Combination_LT_Rep5	GSE271325	GSM8374592	MDA-MB-468 xenograft growing in female NSG, treated with everolimus+SNX631 combination for 148 days
#047	MDA-MB-468_Combination_LT_Rep6	GSE271325	GSM8374593	MDA-MB-468 xenograft growing in female NSG, treated with everolimus+SNX631 combination for 148 days
#048	MDA-MB-468_Combination_LT_Rep7	GSE271325	GSM8374594	MDA-MB-468 xenograft growing in female NSG, treated with everolimus+SNX631 combination for 148 days
#049	MDA-MB-468_Combination_LT_Rep8	GSE271325	GSM8374595	MDA-MB-468 xenograft growing in female NSG, treated with everolimus+SNX631 combination for 148 days
#050	MDA-MB-468_Combination_LT_Rep9	GSE271325	GSM8374596	MDA-MB-468 xenograft growing in female NSG, treated with everolimus+SNX631 combination for 148 days

Supplementary Information References

1. J. Liang *et al.*, CDK8 Selectively Promotes the Growth of Colon Cancer Metastases in the Liver by Regulating Gene Expression of TIMP3 and Matrix Metalloproteinases. *Cancer Res* **78**, 6594-6606 (2018).
2. S. Zheng *et al.*, SynergyFinder Plus: Toward Better Interpretation and Annotation of Drug Combination Screening Datasets. *Genomics, Proteomics & Bioinformatics* **20**, 587-596 (2022).
3. K. M. Whately *et al.*, Nuclear Aurora-A kinase-induced hypoxia signaling drives early dissemination and metastasis in breast cancer: implications for detection of metastatic tumors. *Oncogene* **40**, 5651-5664 (2021).
4. B. Győrffy, Survival analysis across the entire transcriptome identifies biomarkers with the highest prognostic power in breast cancer. *Comput Struct Biotechnol J* **19**, 4101-4109 (2021).
5. J. Li *et al.*, Mediator kinase inhibition reverses castration resistance of advanced prostate cancer. *J Clin Invest* **134** (2024).
6. Y. Song, L. Kelava, L. Zhang, I. Kiss, Microarray data analysis to identify miRNA biomarkers and construct the lncRNA-miRNA-mRNA network in lung adenocarcinoma. *Medicine (Baltimore)* **101**, e30393 (2022).
7. D. M. Schewe, J. A. Aguirre-Ghiso, ATF6alpha-Rheb-mTOR signaling promotes survival of dormant tumor cells in vivo. *Proc Natl Acad Sci U S A* **105**, 10519-10524 (2008).
8. P. Konieczny *et al.*, Interleukin-17 governs hypoxic adaptation of injured epithelium. *Science* **377**, eabg9302 (2022).
9. V. Ribeiro *et al.*, NFIXing Cancer: The Role of NFIX in Oxidative Stress Response and Cell Fate. *Int J Mol Sci* **24** (2023).
10. B. Wang *et al.*, The Role of the Transcription Factor EGR1 in Cancer. *Front Oncol* **11**, 642547 (2021).
11. P. E. Zage *et al.*, Notch pathway activation induces neuroblastoma tumor cell growth arrest. *Pediatr Blood Cancer* **58**, 682-689 (2012).
12. C. H. Huang *et al.*, ATM Inhibition-Induced ISG15/IFI27/OASL Is Correlated with Immunotherapy Response and Inflamed Immunophenotype. *Cells* **12** (2023).
13. P. Jiang, X. Huo, B. Dong, N. Zhou, X. Zhang, Multi-omics analysis of expression profile and prognostic values of connexin family in LUAD. *J Cancer Res Clin Oncol* **149**, 12791-12806 (2023).

14. B. D. Lehmann *et al.*, Identification of human triple-negative breast cancer subtypes and preclinical models for selection of targeted therapies. *The Journal of Clinical Investigation* **121**, 2750-2767 (2011).
15. R. Z. Morsi, R. Hage-Sleiman, H. Kobeissy, G. Dbaibo, Noxa: Role in Cancer Pathogenesis and Treatment. *Curr Cancer Drug Targets* **18**, 914-928 (2018).
16. G. Palma *et al.*, Interleukin 18: friend or foe in cancer. *Biochim Biophys Acta* **1836**, 296-303 (2013).
17. Y. Oshima *et al.*, Anti-angiogenic action of the C-terminal domain of tenomodulin that shares homology with chondromodulin-I. *J Cell Sci* **117**, 2731-2744 (2004).
18. S. Kolb *et al.*, LRP1B-a prognostic marker in tubo-ovarian high-grade serous carcinoma. *Hum Pathol* **141**, 158-168 (2023).
19. L. Xu *et al.*, SLC1A3 promotes gastric cancer progression via the PI3K/AKT signalling pathway. *J Cell Mol Med* **24**, 14392-14404 (2020).
20. M. Mihelic, C. Teuscher, V. Turk, D. Turk, Mouse stefins A1 and A2 (Stfa1 and Stfa2) differentiate between papain-like endo- and exopeptidases. *FEBS Lett* **580**, 4195-4199 (2006).
21. Y. C. Huang *et al.*, Involvement of ACACA (acetyl-CoA carboxylase α) in the lung pre-metastatic niche formation in breast cancer by senescence phenotypic conversion in fibroblasts. *Cell Oncol (Dordr)* **46**, 643-660 (2023).
22. Z. Jin *et al.*, CRTAC1 enhances the chemosensitivity of non-small cell lung cancer to cisplatin by eliciting RyR-mediated calcium release and inhibiting Akt1 expression. *Cell Death Dis* **14**, 563 (2023).
23. S. Bhattacharyya *et al.*, Autotaxin-lysolipid signaling suppresses a CCL11-eosinophil axis to promote pancreatic cancer progression. *Nat Cancer* **5**, 283-298 (2024).
24. Y. Liu *et al.*, PER3 plays anticancer roles in the oncogenesis and progression of breast cancer via regulating MEK/ERK signaling pathway. *J Chin Med Assoc* **85**, 1051-1060 (2022).
25. T. Ando *et al.*, Tumor-specific interendothelial adhesion mediated by FLRT2 facilitates cancer aggressiveness. *J Clin Invest* **132** (2022).
26. G. B. Kim *et al.*, Quantitative immunopeptidomics reveals a tumor stroma-specific target for T cell therapy. *Science translational medicine* **14**, eabo6135 (2022).
27. X. Xu *et al.*, Pan-cancer integrated analysis of ANKRD1 expression, prognostic value, and potential implications in cancer. *Scientific reports* **14**, 5268 (2024).

28. S. Sanders *et al.*, The Presence and Potential Role of ALDH1A2 in the Glioblastoma Microenvironment. *Cells* **10** (2021).
